# Identification of shared tumor epitopes from endogenous retroviruses inducing high-avidity cytotoxic T cells for cancer immunotherapy

**DOI:** 10.1126/sciadv.abj3671

**Published:** 2022-01-26

**Authors:** Paola Bonaventura, Vincent Alcazer, Virginie Mutez, Laurie Tonon, Juliette Martin, Nicolas Chuvin, Emilie Michel, Rasha E. Boulos, Yann Estornes, Jenny Valladeau-Guilemond, Alain Viari, Qing Wang, Christophe Caux, Stéphane Depil

**Affiliations:** 1 Centre de Recherche en Cancérologie de Lyon (CRCL), UMR INSERM U1052 CNRS 5286, Lyon, France.; 2 Centre Léon Bérard, Lyon, France.; 3 ErVaccine Technologies, Lyon, France.; 4 Synergie Lyon Cancer, Plateforme de bioinformatique « Gilles Thomas », Lyon, France.; 5 CNRS-Institut de Biologie et Chimie des Protéines UMR 5086, Lyon, France.; 6 Complete Omics, Baltimore, MD, USA.; 7 Université Claude Bernard Lyon 1, Lyon, France.

## Abstract

Human endogenous retroviruses (HERVs) represent 8% of the human genome. HERV products may represent tumor antigens relevant for cancer immunotherapy. We developed a bioinformatic approach to identify shared CD8^+^ T cell epitopes derived from cancer-associated HERVs in solid tumors. Six candidates among the most commonly shared HLA-A2 epitopes with evidence of translation were selected for immunological evaluation. In vitro priming assays confirmed the immunogenicity of these epitopes, which induced high-avidity CD8^+^ T cell clones. These T cells specifically recognize and kill HLA-A2^+^ tumor cells presenting HERV epitopes on HLA molecules, as demonstrated by mass spectrometry. Furthermore, epitope-specific CD8^+^ T cells were identified by dextramer staining among tumor-infiltrating lymphocytes from HLA-A2^+^ patients with breast cancer. Last, we showed that HERV-specific T cells lyse patient-derived organoids. These shared virus-like epitopes are of major interest for the development of cancer vaccines or T cell–based immunotherapies, especially in tumors with low/intermediate mutational burden.

## Teaser

A bioinformatic approach identifies tumor epitopes from HERVs inducing high-avidity CD8^+^ T cells cytotoxic against tumor cells.

## INTRODUCTION

The adaptive T cell immune response in cancer relies on the recognition of tumor epitopes specifically expressed by tumor cells (*1*). The role of neoantigens, generated by nonsynonymous mutations specific to the tumor genome, has been extensively studied in the past decade (*2*), and many clinical trials testing combinations of neoantigens in personalized cancer vaccines have been initiated, with encouraging preliminary results (*3*). However, determining the optimal combination of neoepitopes for each patient remains challenging. Furthermore, many tumors are characterized by a low or moderate tumor mutational burden. Therefore, unveiling other families of tumor antigens, such as those derived from splice variants, fusion proteins, or endogenous retroelements, possibly shared among different cancer subtypes, is of utmost importance for the development of off-the-shelf therapies in solid tumors (*4*).

Human endogenous retroviruses (HERVs) represent 8% of the human genome (*5*). Although most HERV genes are nonfunctional due to DNA recombination, mutations, and deletions, some produce functional proteins including the group-specific antigen (Gag), polymerase (Pol) with reverse transcriptase, and the envelope (Env) surface unit (*6*). Most HERVs are silenced by epigenetic mechanisms in normal cells (*7*). HERVs are unmethylated and aberrantly expressed in some tumors, including breast cancer, ovarian cancer (*8*), prostate cancer (*9*), and melanoma (*10*). HERVs were reported to be possible pathogenic agents in carcinogenesis through their involvement in insertional mutagenesis, chromosomal aberrations, or long terminal repeat–induced oncogene activation (*6*, *11*). Furthermore, some HERV proteins, such as HERV-K Rec and Np9, are also putative oncogenes (*6*).

HERVs may represent an interesting source of shared tumor antigens (*5*). Because of their homology with “non-self” viral antigens and their limited expression in normal tissues, some HERVs may induce efficient cytotoxic T cell (CTL) responses in the absence of negative selection of reactive T cells in the thymus (*12*). A study reporting a CD8^+^ T cell response against an ERVK3-derived epitope in a patient with melanoma was published by Schiavetti *et al.* (*13*) in 2002. Since this first report, other studies have shown that HERVs can induce T cell responses in patients with cancer, notably in renal cell carcinoma (*14*, *15*), colorectal cancer (*16*), seminoma (*17*), and breast cancer (*18*). However, very few studies have thus far identified HERV-derived T cell epitopes and characterized the corresponding T cell clones. The large number of HERVs integrated in the genome and the associated polymorphism of these repeated sequences render target discovery and validation very difficult, hampering the development of immune therapy targeting HERV-derived epitopes. We present here an original bioinformatic-based method to identify shared T cell epitopes derived from HERV viral antigens specifically overexpressed in different cancer subtypes. We show that these HERV-derived epitopes efficiently prime antitumor CTL clones of high avidity and could thus represent relevant targets for cancer immunotherapy.

## RESULTS

### A machine learning–based approach allows the identification of HERVs associated with CTL response

**Fig. 1.**
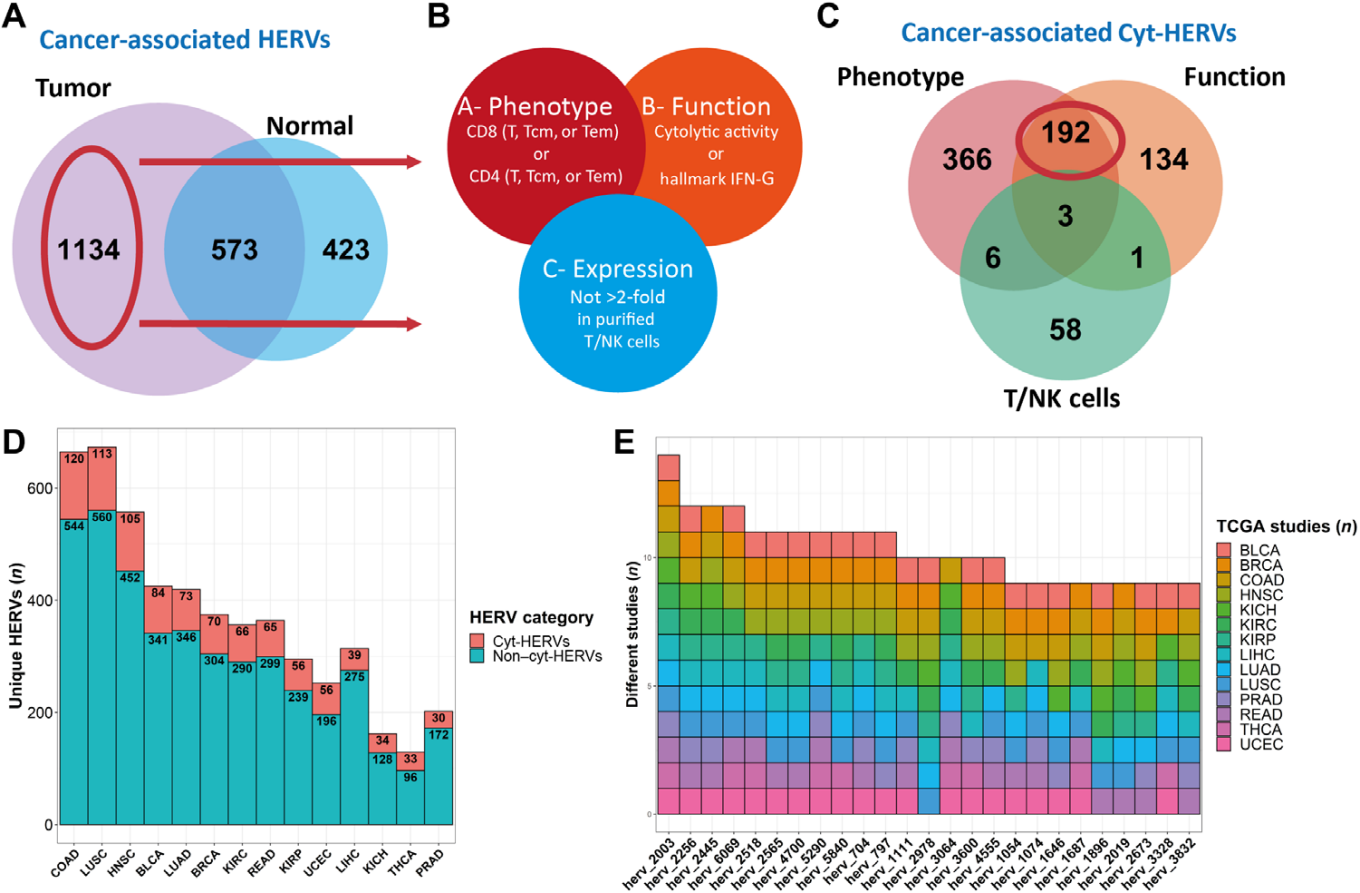
Pancancer identification of HERVs associated with CTL responses. (**A**) Venn diagram representing the total number of HERVs overexpressed in a tumor versus its normal counterpart (peritumoral tissue) and the total number of HERVs overexpressed in a normal peritumoral tissue versus its tumoral counterpart. HERVs overexpressed in at least one tumor and never overexpressed in any peritumoral tissue are considered cancer-associated. (**B**) Venn diagram of the selection criteria for an HERV to be annotated as associated with CTL response (cyt-HERV). Each HERV had to be associated with both a phenotype (CD8 or CD4 T cell signatures) and a function [cytolytic activity (granzyme B and perforin 1) or IFN-γ signature] criteria (A and B) and not overexpressed in normal purified T/NK cells (A and B not C). (**C**) Venn diagram of CAHs’ association with CTL responses criteria defined in (A). A total of 192 HERVs are annotated as cyt-HERVs. (**D**) Proportion of CAHs annotated as cyt-HERVs per cancer subtype. Cyt-HERVs are colored in red. (**E**) The top 25 most shared cyt-HERVs with their respective tumors. The number of different cancers is represented on the *y* axis.

To optimize the epitope detection, we developed a new pipeline for annotating HERVs. For this, we reviewed multiple HERV databases and selected a recent and complete reference of 3173 HERVs, mostly composed of complete proviral sequences, thus having a higher probability of containing translated peptides (*19*). We assessed HERV expression in 8893 primary tumor samples from 29 different cancer types from The Cancer Genome Atlas (TCGA) pancancer RNA sequencing (RNA-seq) database using HervQuant (fig. S1A and table S1) (*15*). We selected cancers with at least 10 available matched peritumoral samples (*n* = 14; table S1) to filter HERVs highly expressed in tumors and not in normal tissue [cancer-associated HERVs (CAHs)]. Differential HERV expression analysis unveiled 1134 CAH candidates (Fig. 1A). To reduce the number of candidates to test, we next applied a second filter to retain only HERVs associated with a CTL response among CAHs (cyt-HERVs). Cyt-HERV annotation was based on two inclusion criteria, namely, the association of each HERV with at least one CD4 or CD8 T cell phenotype (A) and function (B) signature, and one exclusion criterion, namely, its expression by purified T or natural killer (NK) cells (C) (A and B not C) (Fig. 1B). To reduce the risk of false-positive association and control for the high collinearity encountered with HERV expression, these associations were evaluated by L1 penalized regression (*20*) to retain only HERVs that are highly associated with CTL responses, controlling for cancer subtypes. A machine learning–based approach was used to test the associations independently for each cancer type (see Materials and Methods for full details), leading to the final identification of 192 cyt-HERVs (Fig. 1C). Sub-cancer analysis revealed that colon adenocarcinoma (COAD), lung squamous cell carcinoma (LUSC), head and neck squamous cell carcinoma (HNSC), bladder urothelial carcinoma (BLCA), and lung adenocarcinoma (LUAD) were the top five cancers with the highest total number of cyt-HERVs (Fig. 1D).

Overall, cyt-HERVs constituted around 15% of the total CAHs, greatly reducing the number of potential candidates. Among the most shared cyt-HERVs, 11 were overexpressed in more than 10 different types of cancers, including 3 HERVs (herv_2256, herv_6069, and herv_4700) formerly reported to induce CD8^+^ T cell responses (*10*, *14*, *15*) (Fig. 1E). Analysis of the mean β value of the 10 nearest surrounding probes from TCGA Illumina 450k methylation data revealed that more cyt-HERVs significantly correlated with local demethylation (*n* = 37) than methylation (*n* = 15), suggesting a partial epigenetic control of these HERVs (fig. S1B).

### Selection of conserved Gag and Pol HERV-K/HML-2 motifs among cyt-HERVs leads to the identification of shared CD8^+^ T cell epitopes

**Fig. 2.**
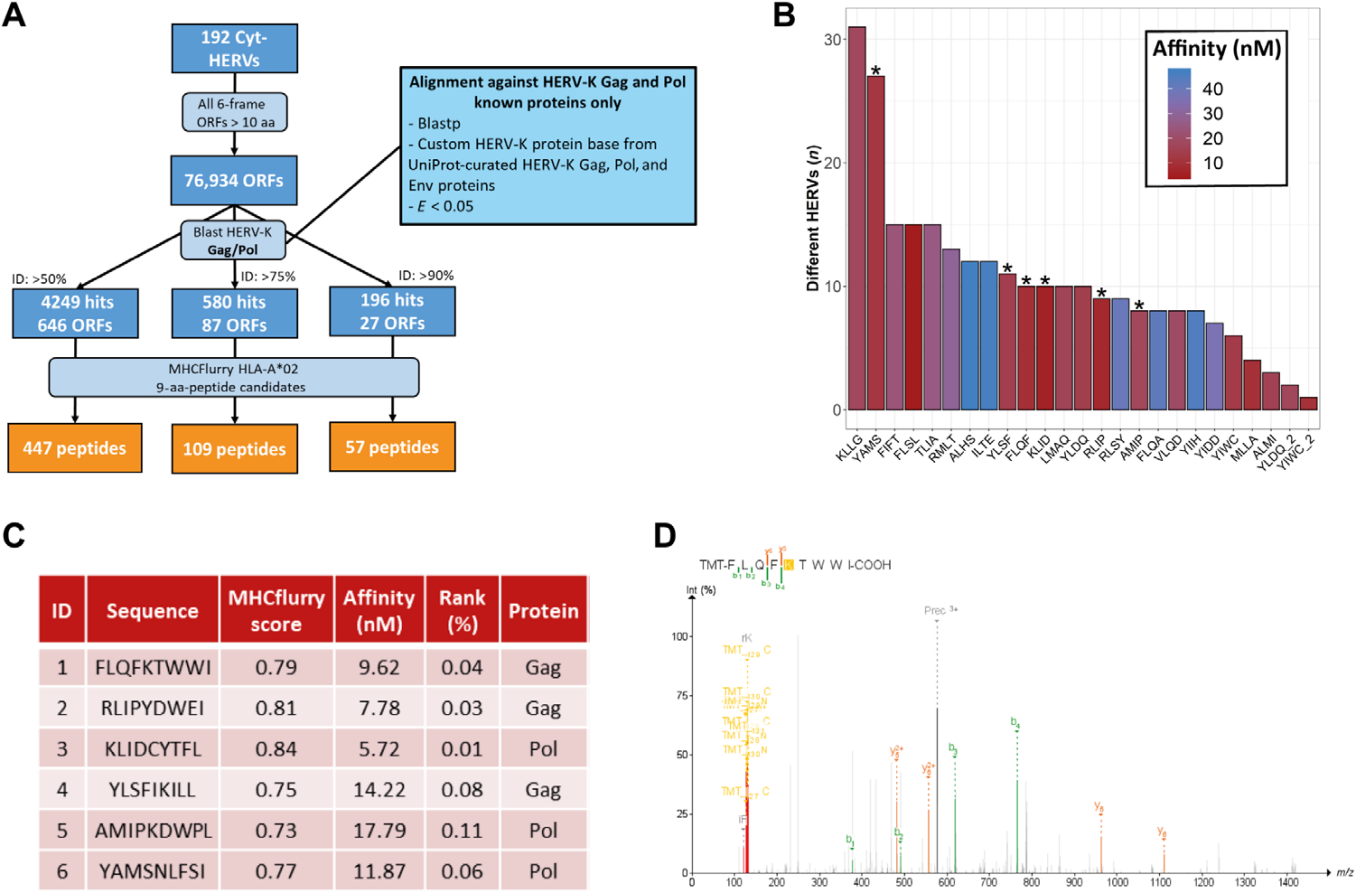
Selection of shared HLA-A2 epitopes derived from Gag and Pol HERV-K/HML-2. (**A**) Flow chart of peptide selection from cyt-HERV sequences. aa, amino acid. (**B**) Bar chart of the top 25 most shared peptides predicted as strong HLA-A*02 binders among the 192 cyt-HERVs. Selected peptides (P1 to P6) are marked with a star. (**C**) Characteristics of the six selected HLA-A2 epitopes. Nine-mer peptides were selected according to their predicted HLA-A*02 affinity (considering strong binders for percentile ranks of ≤0.5) and the number of HERVs containing their sequences. (**D**) MS/MS detection of P1 epitope in a sample from the CPTAC breast cancer prospective dataset. MS/MS spectrum is identified by Pepquery analysis (Peptide Spectrum Match, *P* value of 0.0063). *m/z*, mass/charge ratio.

We next assessed the presence of shared T cell epitopes among these cyt-HERVs, focusing on human lymphocyte antigen A2 (HLA-A2), the most common HLA class I allele (*21*). We translated our 192 cyt-HERV sequences into the six possible frames and retained predicted open reading frames (ORFs) of at least 10 amino acids. To reduce the number of false positives (nontranslated sequences), we aligned these ORFs against known HERV-K/HML-2 Gag and Pol proteins referenced in UniProt and kept only ORFs with 90% homology with known existing HML-2 proteins (*22*). This conservative approach led to the identification of 57 HML-2 HLA-A*0201 epitope candidates from 27 distinct ORFs (Fig. 2A), with herv_2410 and herv_6069 showing the highest number of conserved HML-2–derived ORFs (fig. S2A). To better appreciate the distribution of these epitopes, we relocated each peptide among all the CAHs. The top 25 most shared epitopes are shown in Fig. 2B. Thirteen unique epitopes were present in at least 10 different HERVs (Fig. 2B). For further biological validation and immunological assays, we selected six of the most shared epitope candidates: three from Gag (P1, P2, and P4) and three from Pol (P3, P5, and P6) (Fig. 2C). Analysis of mass spectrometry (MS) data from TCGA and Clinical Proteomic Tumor Analysis Consortium (CPTAC) (*23*) showed evidence of translation for P1, P2, P3, P5, and P6 peptides (Fig. 2D and fig. S2B). P4 was also selected as it had been described among HLA-I eluted peptides from tumors (patent WO2019/162110A1). Alignment against the human proteome revealed that the sequences of these epitope candidates did not match any self-protein sequence (table S2). Notably, these HLA-A2 epitopes were not predicted as strong binders for the other most common HLA-A and HLA-B alleles (table S3).

### Triple-negative breast cancer is characterized by many cyt-HERVs containing shared HERV epitopes

**Fig. 3.**
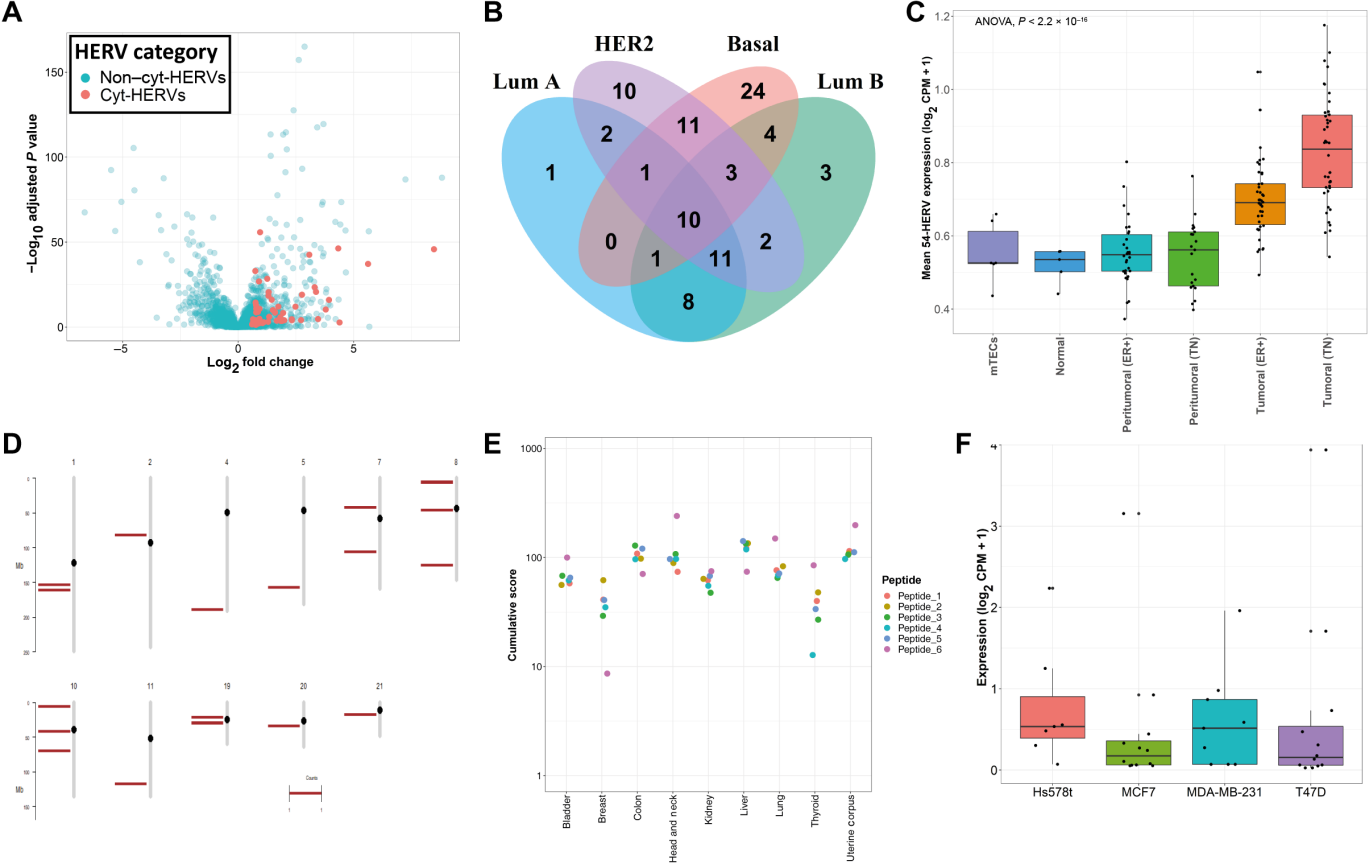
Shared CD8^+^ T cell epitopes derived from conserved Gag and Pol HERV-K/HML-2 motifs are expressed in TNBC. (**A**) Volcano plot of HERVs overexpressed in basal breast cancer subtype compared to peritumoral samples in TCGA database. cyt-HERVs are represented in red. (**B**) Venn diagram of the total number of cyt-HERVs overexpressed in each subtype of breast cancer in TCGA database. (**C**) Mean expression of the 54 cyt-HERVs overexpressed in TCGA basal subtype, in the independent database of Varley *et al.* (*25*), and in mTECs. (**D**) Chromosomal location of the 18 peptide-containing HERVs overexpressed in the basal breast cancer subtype. Each bar corresponds to an HERV locus. (**E**) Cumulative expression score of the epitope-containing HERVs in TNBC versus normal tissues. This score was calculated for each epitope and each available peritumoral sample. (**F**) Expression of the 18 peptide-containing CAHs in the breast cancer basal (Hs578t and MDA-MB-231) and luminal A (MCF7 and T47D) cell lines analyzed by riboseq.

Owing to the well-characterized expression of HERVs in triple-negative breast cancer (TNBC) (*24*) and the availability of an RNA-seq database comprising normal samples (*25*), we then focused on breast cancer. Differential HERV expression analysis uncovered a total of 497 CAHs expressed across different breast cancer subtypes, among which 91 were annotated as cyt-HERVs (Fig. 3, A and B). Fifty-four of these 91 cyt-HERVs were expressed in the basal subtype (Fig. 3B). The mean expression of these 54 cyt-HERVs was significantly higher in TNBC and ER^+^ (estrogen receptor–positive) samples compared to peritumoral or normal breast tissues from an independent dataset (Fig. 3C). Analysis of the mean expression level of these 54 HERVs in medullary thymic epithelial cells (mTECs) revealed a similar profile as in normal tissues (Fig. 3C), suggesting that the thymus may not induce a negative selection of the corresponding HERV antigen-specific T cells. We confirmed the high expression of these 54 cyt-HERVs in breast cancer cell lines sequenced in Varley *et al.*’s (*25*) study (fig. S3A) and in cell lines from the Broad Institute Cancer Cell Line Encyclopedia (fig. S3B). The top 25 most shared epitope candidates among all CAHs expressed in the basal subtype contained the six previously identified peptides P1 to P6 (fig. S3C).

We next selected HERVs containing the sequences of the six previously identified epitope candidates P1 to P6 among the CAHs expressed in the basal subtype. Eighteen different CAHs contained at least one of the six peptides in their ORFs (Table 1). Genomic mapping of the corresponding loci showed a diffuse location for these 18 HERVs on chromosomes (Fig. 3D). To quantify the differential expression of the epitope-containing HERVs in TNBC versus normal tissues (represented here by each available peritumoral sample), we used the π value score that takes into account both statistical significance, given by the *P* value, and biological significance expressed by the fold change (*26*). A cumulative expression score was then calculated for each epitope by summing the π values of all the HERVs containing its sequence (Fig. 3E). This score was between 10 and 200 in most cases, which confirmed the significant overexpression of the epitope-containing HERVs in TNBC versus each evaluated normal sample. Last, analysis of ribosome profiling (riboseq) data from a previously published study (*27*) revealed evidence of translation for the 18 peptide-containing CAHs in four different breast tumor cell lines including two basal and two luminal A subtypes (Fig. 3F). Overall, our bioinformatic approach allowed us to select a limited number of HERV-derived T cell epitopes specifically overexpressed by tumor cells and most likely to be immunogenic among a large number of HERV candidates.

**Table 1.** Peptide-containing HERVs overexpressed in the basal breast cancer subtype. Previously identified peptides 1 to 6 have been located among HERVs overexpressed in the basal breast cancer subtype in TCGA patients. Log_2_ fold changes have been calculated and shrunk using DESEQ2. *P*_adj_, adjusted *P* value; FC, fold change.

***herv_id***	**baseMean**	**Log_2_ FC**	**FC**	***P*_adj_**	**P1**	**P2**	**P3**	**P4**	**P5**	**P6**
*herv_2953*	1.42	3	7.99	6.14 × 10^−17^						x
*herv_4833*	507.59	2.4	5.28	2.84 × 10^−128^	x	x				x
*herv_3232*	144.11	2.01	4.02	2.33 × 10^−29^						x
*herv_4873*	32.5	1.56	2.94	4.04 × 10^−25^			x	x	x	x
*herv_6069*	677.18	1.31	2.48	3.27 × 10^−19^	x	x	x	x	x	x
*herv_2025*	8.18	1.21	2.32	1.05 × 10^−11^	x	x	x	x	x	x
*herv_6079*	35.73	1.16	2.23	2.28 × 10^−30^		x	x	x	x	x
*herv_2704*	28.63	1.1	2.14	3.63 × 10^−16^	x	x	x	x	x	x
*herv_1741*	24.46	0.93	1.9	1.70 × 10^−14^			x	x		x
*herv_3192*	15.98	0.87	1.82	5.01 × 10^−22^	x	x		x		x
*herv_3288*	24.52	0.83	1.78	1.92 × 10^−18^						x
*herv_2794*	8.4	0.77	1.7	1.49 × 10^−13^					x	
*herv_2288*	32.98	0.71	1.64	1.79 × 10^−8^						x
*herv_2582*	8.63	0.69	1.61	9.33 × 10^−9^	x	x	x	x	x	x
*herv_4679*	157.18	0.69	1.61	9.88 × 10^−29^						x
*herv_2476*	415.1	0.64	1.56	1.64 × 10^−16^						x
*herv_4695*	30.94	0.6	1.51	4.43 × 10^−11^	x	x	x	x	x	x
*herv_3652*	74.36	0.6	1.51	1.61 × 10^−13^	x	x	x		x	x

### HERV-derived epitopes induce strong and polyfunctional T cell responses

**Fig. 4.**
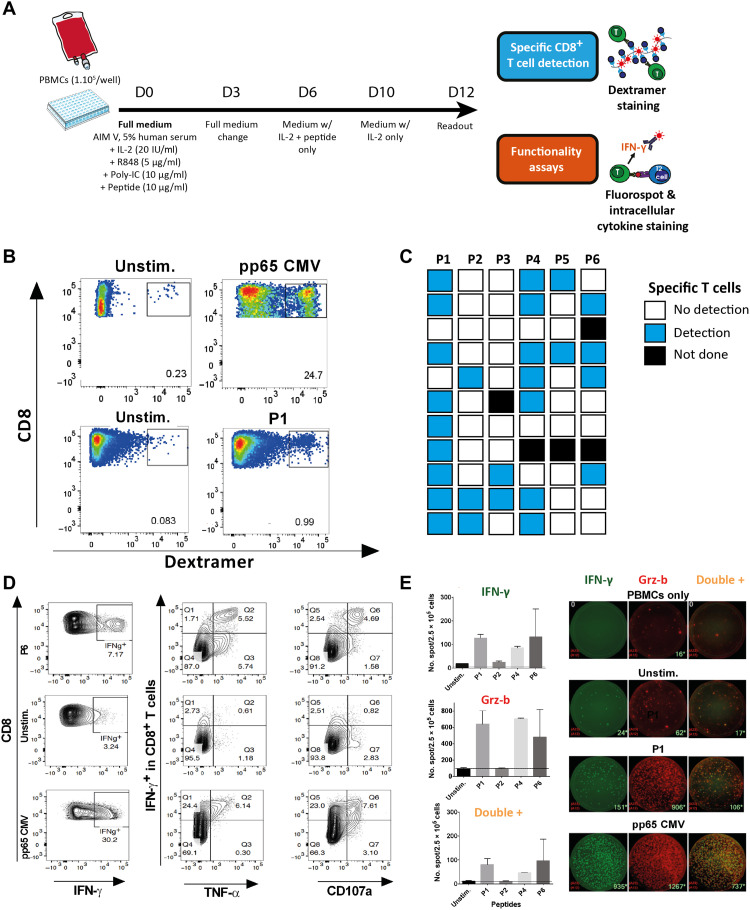
HERV-derived epitopes induce polyfunctional CD8^+^ T cell responses. (**A**) Schematic representation of the in vitro priming protocol. (**B**) Representative example [healthy donor 2 (HD2)] of dextramer staining of peptide-stimulated (bottom right) and unstimulated (bottom left) PBMCs gated on the CD8^+^ T cells. CMV pp65–stimulated (top right) and unstimulated (top left) PBMCs are used as controls. (**C**) Summary of the results obtained with PBMCs from 11 HLA-A2–positive HD (HD1 to HD11, one donor per line). (**D**) Plots of IFN-γ (left panels), IFN-γ and tumor necrosis factor–α (TNF-α) (center panels), or IFN-γ and CD107a (right panels) staining gated on CD8^+^ T cells. PBMCs were stimulated with peptide (here P6, top line), no peptide (central line,) or CMV pp65 peptide (bottom line). Plots (HD5) are representative of the data summarized in fig. S4E. (**E**) Mean number and SD of IFN-γ^+^, Grz-b^+^, and double IFN-γ^+^ Grz-b^+^ spots counted on Fluorospot after stimulation of HLA-A2–positive PBMCs with different peptides (duplicates). Representative wells (HD2) are shown on the right: IFN-γ, Grz-b, and double-positive spots for PBMCs alone and 10:1 PBMC:T2 cocultures using either unstimulated PBMCs, P1-stimulated PBMCs, or CMV pp65–positive control PBMCs. pp65, phosphoprotein 65; Unstim, unstimulated.

We then evaluated the capacity of the selected epitope candidates to induce efficient T cell responses. The HLA-A2 affinity of the six selected peptides was first confirmed using an in vitro binding assay on purified HLA-A*02:01 molecules (fig. S4A). To assess the immunogenicity of these six peptides, we developed an optimized in vitro priming assay performed on peripheral blood mononuclear cells (PBMCs) from HLA-A2–positive donors (see Fig. 4A and Materials and Methods for details). The dextramer-based quantification of peptide-specific CD8^+^ T cells (gating strategy; fig. S4B) revealed the presence of specific T cells for all peptides, with variations among donors (Fig. 4, B and C, and fig. S4C). P1 appeared to be the most immunogenic peptide with significant T cell responses in 9 of 11 donors, followed by P4 (7 of 10), P6 (4 of 9), and P2 (3 of 11) (Fig. 4C). The immunogenicity of these peptides was further confirmed by a classical assay using monocyte-derived dendritic cells (MoDCs) prepared from five HLA-A2–positive healthy donors (fig. S4D). Flow cytometry analysis showed a CD8^+^ T cell interferon-γ (IFN-γ) production when peptide-stimulated PBMCs were cocultured with T2 cells pulsed with the cognate epitopes (fig. S4, B and E). Notably, P1 also induced the highest IFN-γ response compared to the other peptides. In agreement with the bioinformatic prediction, no specific T cell induction was observed using PBMCs from HLA-A2–negative donors (*n* = 5) (fig. S4F).

On the basis of these results, we selected P1, P2, P4, and P6 for further experiments. A polyfunctional IFN-γ^+^ TNF-α^+^–specific CD8^+^ T cell response was observed upon coculture of stimulated PBMCs with peptide-pulsed T2 cells, associated with the presence of the degranulation marker CD107a (Fig. 4D). Fluorospot assay under the same coculture conditions confirmed the secretion of IFN-γ and granzyme B with the presence of double-positive cells (Fig. 4E).

To confirm that the selected epitopes can be efficiently processed from the native HERV sequence by antigen-presenting cells, 29- to 34-mer synthetic long peptides (SLPs), corresponding to the native Gag or Pol polypeptide sequence and containing P1, P2, P4, or P6 epitopes, were synthesized and used in an MoDC-based priming assay. A dextramer readout confirmed the induction of CD8^+^ T cells specific for all epitopes except P4 after the use of the corresponding SLP (fig. S4G).

### Epitope-specific CD8^+^ T cell clones are characterized by T cell receptors of high predicted affinity

**Fig. 5.**
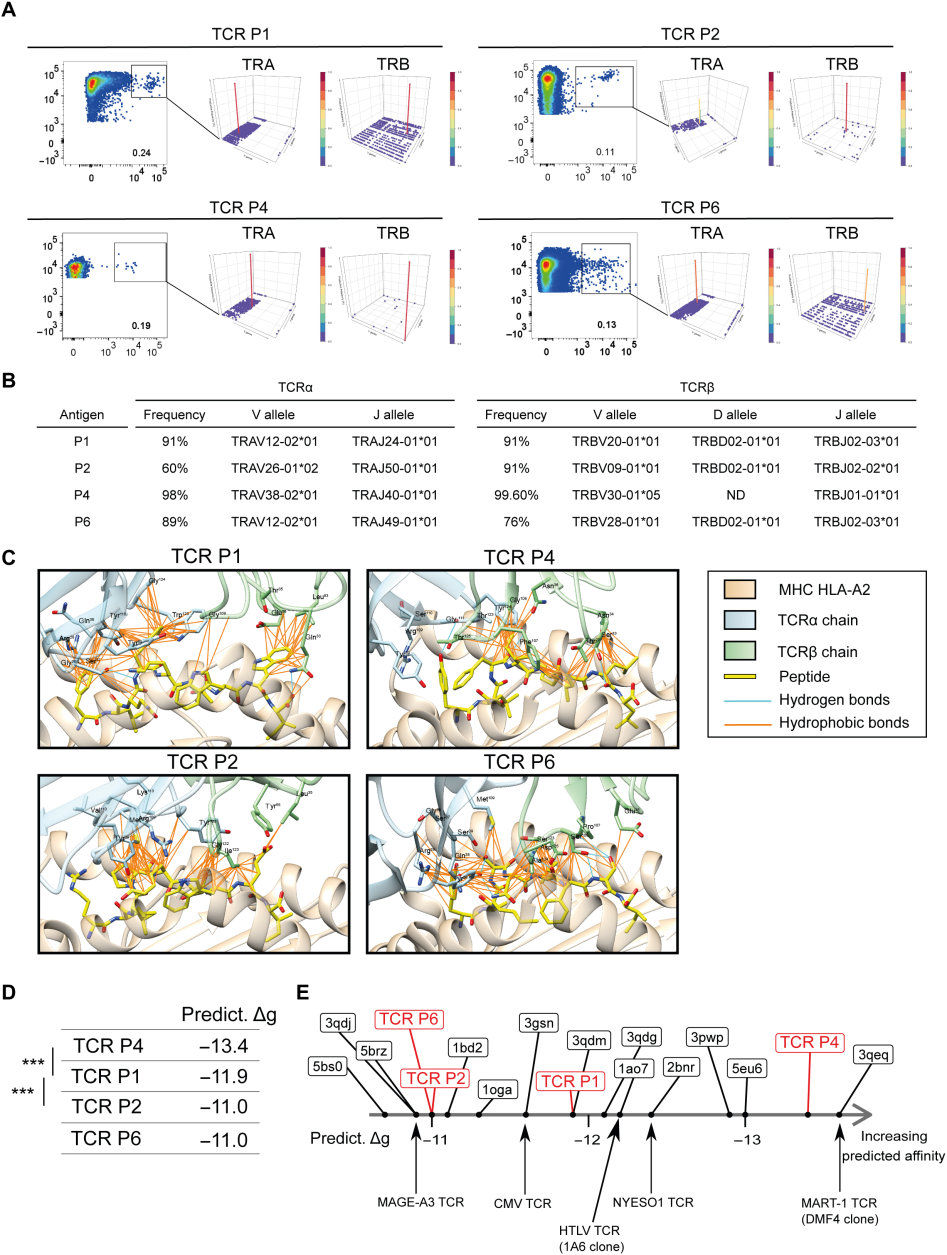
Visualization of CDR loops and CDR loop interactions with peptides. (**A**) Epitope-specific CD8^+^ T cells from HLA-A2–positive healthy donors (P1 from HD22, P2 and P4 from HD11, and P6 from HD23) sorted by fluorescence-activated cell sorting. Sorted cells (left in all four panels, gated) underwent TCR sequencing, and results are shown in Manhattan plot reports of the V/J recombination of the TCRα (center in all panels) and TCRβ (right in all panels). V and J segments are represented according to chromosomal location on the *x* and *y* axis, respectively. Productive frequencies of clones are represented on the *z* axis. (**B**) Productive frequency of the TCRα and TCRβ CDR3 sequences for the top clones specific to each peptide (P1, P2, P4, and P6) and the corresponding resolved V, D, and J alleles. ND, not determined. (**C**) 3D representative models of the TCR-pMHC interface for each identified TCR. The MHC chains (HLA-A2) are colored in beige, TCRα chains in light blue, TCRβ chains in light green, and peptides in yellow. Peptide and TCR residues involved in the TCR-pMHC interaction are shown as sticks, with O atoms in red, N atoms in dark blue, and S atoms in yellow. Hydrogen bonds are represented as cyan lines, salt bridge as red lines, and hydrophobic contacts as orange lines. (**D**) Predicted binding affinities (Predict. Δg) are expressed in kilocalories per mole. Average values are reported. Stars indicate significant statistical test (Welch two-sample *t* test) at the 5% level. (**E**) Diagram ranking of modeled HERV-specific TCR-pMHC and reference TCR-pMHC complexes available in the Protein Data Bank and obtained from crystallography data, according to their predicted binding affinity. CDR, complementarity-determining region; TRA, α chain of TCR; TRB, β chain of TCR.

P1-, P2-, P4-, and P6-specific CD8^+^ T cells were sorted by flow cytometry using dextramer staining and expanded on feeder cells (see Materials and Methods). More than 90% (90 to 99%) of the CD8^+^ T cells were dextramer positive after one (P1) or two steps (P2, P4, and P6) of selection expansion (fig. S5A). T cell receptor β (TCRβ) immunosequencing confirmed the presence of dominant clones with a unique Vβ rearrangement representing 90.8, 90.7, 99.6, and 76% of the expanded T cells for P1, P2, P4, and P6, respectively (Fig. 5, A and B). Notably, the V/D/J recombination sequences of TCRβ characterizing these clones were not present in the T cell bulk before peptide stimulation (threshold sensitivity, 3 × 10^−6^). TCRα chains were also sequenced and confirmed the presence of a unique major clone for P1, P4, and P6, enabling TCR pairing and modeling. Because two major Vα rearrangements were obtained for P2, the predominant rearrangement occurring at 60% frequency was used for TCR modeling.

The affinity of the T cell clones specific for the peptides P1, P2, P4, and P6 was then characterized by considering three-dimensional (3D) models of the TCR–peptide–major histocompatibility complex (pMHC) complexes (see Materials and Methods). The stability of macromolecular complexes is due to the formation of favorable interactions at the interface such as hydrogen bonds, salt bridges, and hydrophobic interactions. Figure 5C displays these favorable interactions in each of the 3D models of TCR-pMHC complexes. These interactions involve specific side chains of the peptides that are exposed at the TCR-pMHC interface. In the TCR P1 complex, Phe^1^, Phe^4^, and Trp^8^ side chains of the peptide form several hydrophobic interactions, and the backbone atoms of Phe^4^ and Ile^9^ are involved in H-bonds. In the TCR P2 complex, several hydrophobic interactions are mediated by peptide residues Pro^4^, Tyr^5^, and Trp^7^. In TCR P4, peptide residues Ile^5^, Ile^7^, and Leu^8^ form several hydrophobic interactions, while Tyr^1^ and Lys^6^ side chains, as well as Phe^4^ and Leu^8^ backbone atoms, are involved in H-bonds. In TCR P6, Tyr^1^, Ser^4^, Asn^5^, Leu^6^, and Phe^7^ form several hydrophobic interactions, while Ser^4^/Leu^6^/Ser^8^ backbones and Tyr^1^/Ser^4^/Asn^5^/Ser^8^ side chains form eight H-bonds.

Overall, this analysis of the predicted 3D models suggests that the TCR-pMHC complexes are stabilized by several favorable noncovalent interactions, supporting the notion that the TCRs identified after clonal expansion of HERV-specific T cells form a stable complex with the peptides presented by HLA-A2 molecules. To gain further insight, we submitted the 3D models to binding affinity prediction (Fig. 5D). When compared to reference TCR-pMHC complexes available in the Protein Data Bank (which were obtained from crystallography data), the predicted affinities of the identified TCRs match clinically relevant TCR affinities, such as TCRs targeting MAGE-A3, NY-ESO-1, MART-1, human T cell leukemia virus, or cytomegalovirus (CMV) (Fig. 5E). Hence, the HERV-specific TCRs identified are predicted to stably interact with their respective pMHC complexes, reminiscent of high-affinity TCRs.

### High-avidity HERV-specific T cell clones recognize and kill tumor cells

**Fig. 6.**
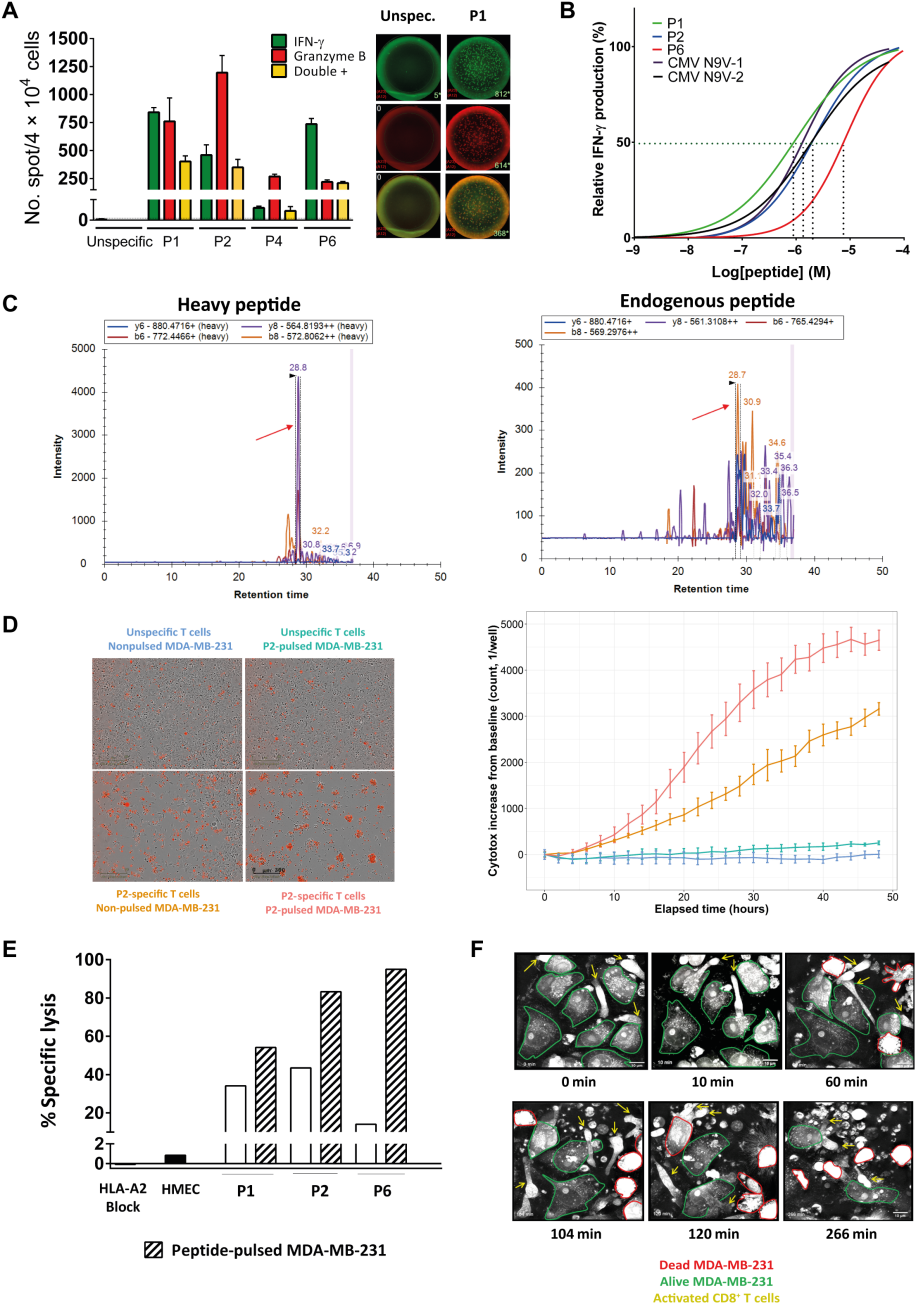
HERV-specific T cell clones are functional and recognize and kill tumor cells. (**A**) Left: Mean number and SD of IFN-γ, Grz-b, and double-positive spots counted on Fluorospot (duplicates). Right: Representative wells of IFN-γ (green), Grz-b (red), and double-positive (yellow) spots for unspecific (dextramer-negative, left) and peptide-specific (here P1) T cells (right) cocultured with T2 cells in a ratio of 10:1 for 24 hours. (**B**) Functional avidity of CD8^+^ T cell clones calculated as nonlinear fit of normalized IFN-γ production. N9-V1 and N9-2: CMV-specific T cell clones (see Materials and Methods). EC_50_ values are represented for each clone by the interpolation of the dashed lines with the *x* axis. (**C**) Valid-NEO transitions of peptide P1 (FLQFKTWWI) are shown on the chromatogram. Absolute quantification was performed as described in (*62*). (**D**) Left: Representative 10× images of cocultures of T cells (here, P2-specific) with MDA-MB-231 cell line (E:T = 2:1) in a 48-hour cell killing assay (IncuCyte). Dead MDA-MB-231 cells are depicted in red. Right: Cell death quantification represented as fluorescence intensity increase from the baseline (*y* axis) as function of the time (hours, *x* axis). The color code is the same as on the left. (**E**) Specific tumor cell lysis at 48 hours. Mean percentage of technical triplicates is plotted for each condition (data representative of at least two independent experiments). (**F**) Pictures (60×) of MDA-MB-231 cocultured with P1-specific CD8^+^ T cells (E:T = 10:1) at different time points (Nanolive). T cells are shown by yellow arrows.

The functionality of the sorted and expanded epitope-specific CD8^+^ T cells was confirmed by Fluorospot using peptide-pulsed T2 cells. A dual IFN-γ and granzyme B secretion was shown, except for the P4-specific T cell clone that appeared to be poorly functional (notably in terms of IFN-γ production) and was then excluded from further analyses (Fig. 6A). The functional avidity was subsequently assessed by loading T2 cells with decreasing concentrations of the cognate peptide (ranging from 10^−4^ to 10^−9^ M) and measuring the lowest peptide concentration necessary to provoke IFN-γ responses in 50% of cells [defined as half-maximal effective concentration (EC_50_)]. The EC_50_ values—estimated at 6.6 × 10^−7^ M, 1.9 × 10^−6^ M, and 6.8 × 10^−6^ M for P1-, P2-, and P6-specific T cells, respectively—are in the same order of magnitude as neoepitope-specific T cell clones (*28*) and CMV-specific T cells (1.2 × 10^−6^ and 1.9 × 10^−6^ for N9V-1 and N9V-2, respectively), suggesting an absence of negative selection in the thymus of the most reactive T cell clones (Fig. 6B).

We next assessed the capacity of these HERV epitope–specific CD8^+^ T cells to recognize and kill tumor cells. We selected as a target candidate the HLA-A2–positive MDA-MB-231 basal BRCA tumor cell line, previously shown to express HERVs containing epitope sequences (Fig. 3F and fig. S3B). To provide evidence that the epitopes are actually presented on the cell surface, an MS-based method was used to analyze peptides eluted from HLA molecules. P1 and P6 epitopes were clearly detected by MS. On the basis of comparison with the heavy isotope–labeled control, we estimated that there were 1.8 copies of P1-HLA complexes on MDA-MB-231 cell surface (Fig. 6C and fig. S6A).

Tumor cells were cocultured with the epitope-specific T cells or with the dextramer-negative CD8^+^ T cell fraction sorted and expanded under the same conditions (negative controls). Flow cytometry analysis highlighted IFN-γ production by approximately 25% of epitope-specific T cells in contact with MDA-MB-231, with a significant increase (>6-fold) compared to the background observed with nonspecific T cells. This IFN-γ production was inhibited by an HLA-A2–blocking monoclonal antibody, demonstrating that the T cell clones specifically recognized the tumor cells in an HLA-A2–restricted manner (fig. S6, B and C).

To monitor tumor cell death in real time, we performed an immune cell killing assay using the IncuCyte technology. T cell clones induced a significant and HLA-A2–restricted killing of MDA-MB-231 cells, as shown by the time-dependent increase in the amount of Cytotox fluorescent reagent of target cells. In contrast, the dextramer-negative fraction of T cells did not induce significant cell death of MDA-MB-231 cells (pulsed or not with the peptide) (Fig. 6D). Specific lysis [at effector-to-target (E:T) ratio = 2:1] was calculated on the basis of the quantification of target cell death after 48 hours after subtracting the alloreactive background (assessed by the target cell death induced with the corresponding dextramer-negative T cell fraction) (see Materials and Methods). A particularly high specific lysis of the tumor cells was achieved with P1- and P2-specific T cells (35 and 44%, respectively), with a more moderate lysis (15%) with P6-specific T cells. The specific lysis was further increased when the target tumor cells were pulsed with the cognate epitope, reaching 55, 80, and even 95% for P1-, P2-, and P6-specific T cells, respectively. Notably, epitope-specific T cell clones did not kill HLA-A2–positive human mammary epithelial cells (HMECs), used here as a negative, normal cell, control (Fig. 6E). These results were further validated using the 3D microscopy Nanolive technology, showing morphological signs of activation of specific T cells associated with killing of most of the tumor cells after 4.5 hours (E:T = 10:1). Again, no cell death was observed when the specific T cells were cocultured with HMECs and when MDA-MB-231 cells were cocultured with nonspecific T cells (Fig. 6F and movies S1 to S3). Similar results were obtained using the TNBC HLA-A2–positive cell line HCC1599 as target (movies S4 and S5 showing signs of specific T cell activation with morphological changes of the multicellular aggregates formed by the target). Together, these data show that the selected epitopes elicit high-avidity CD8^+^ T cell clones that selectively recognize and kill HERV-expressing tumor cells.

### HERV-specific T cells are present among tumor-infiltrating T cells

**Fig. 7.**
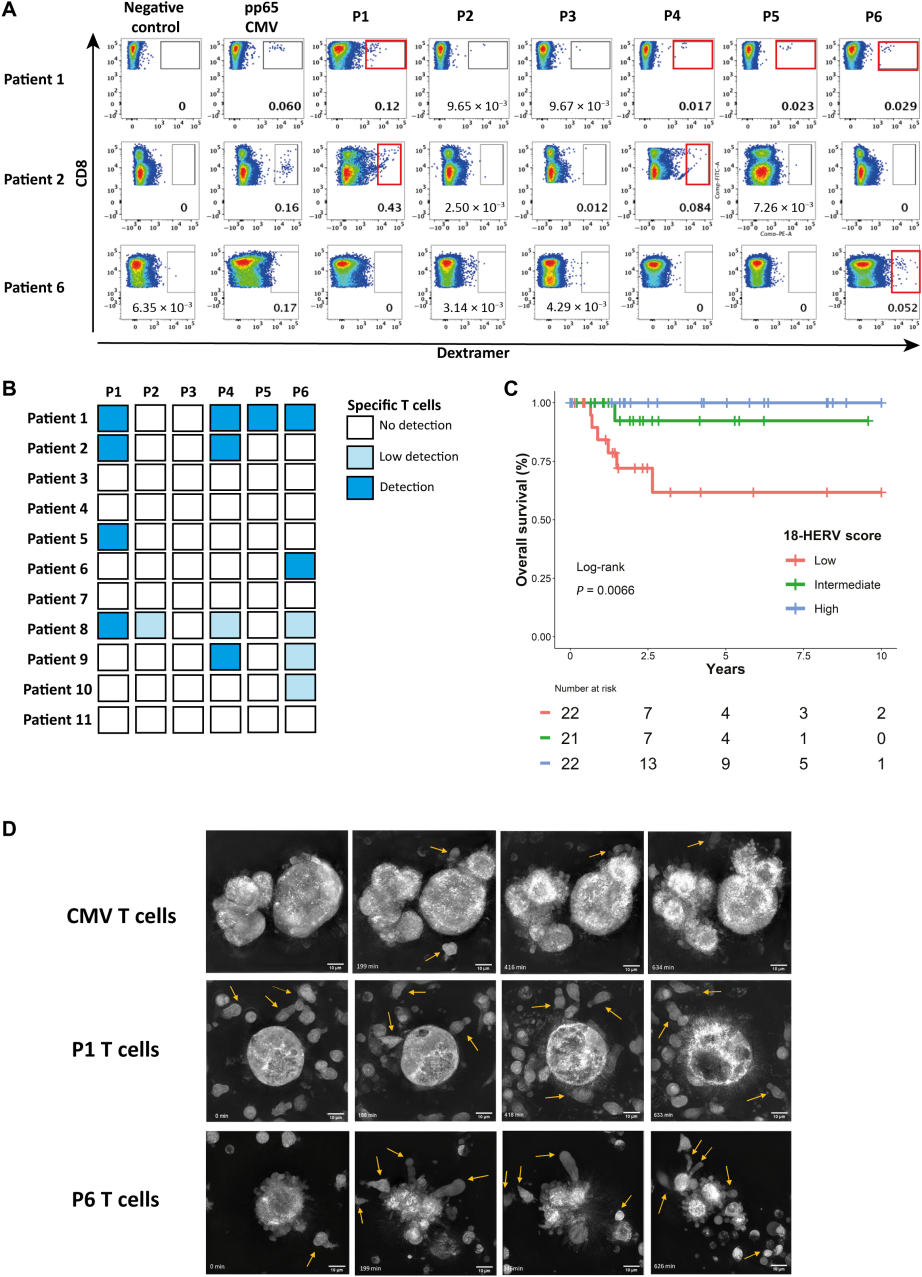
HERV-specific T cells are present among tumor-infiltrating T cells. (**A**) Representative panels of dextramer staining for the six HERV epitopes in TILs from three HLA-2–positive patients with TNBC. Dextramer-positive cells were gated on CD8^+^ T cells. Negative control: Dextramer complexed to a non-natural irrelevant peptide (ALIAPVHAV). (**B**) Result summary of dextramer CD8^+^ T cell detection among TILs from 11 HLA-A2–positive patients with TNBC for the six HERV peptides. (**C**) Overall survival according to 18-HERV score in TCGA HLA-A2 TNBC patients (*n* = 65). Patients were divided in three groups according to the score terciles: blue line, high expression (*n* = 22); green line, intermediate expression (*n* = 21); red line, low expression (*n* = 22). (**D**) Pictures (60×) of TNBC organoids cocultured with CMV-, P1-, or P6-specific CD8^+^ T cell clones (top-down) acquired at different time points using Nanolive technology. T cells are shown by yellow arrows.

To test our hypothesis that an adaptive immune response against HERVs may exist in patients with cancer, we assessed by dextramer staining the presence of HERV epitope–specific T cells among polyclonally expanded tumor-infiltrating lymphocytes (TILs) from HLA-A2 patients with TNBC (without any peptide-specific stimulation). HERV-specific TILs were observed for at least one epitope in 7 of the 11 analyzed tumor samples, with variations in terms of epitope specificity and frequency from one patient to another. P1, P4, and P6 were the most frequently recognized peptides, with a dextramer-based identification in 4 of 11, 4 of 11, and 5 of 11 cases, respectively, whereas no significant staining was seen for P3 (Fig. 7, A and B).

These results prompted us to investigate the potential link between the outcome of patients with TNBC and the expression of the 18 CAHs containing these HLA-A2 epitopes. We established a score based on the mean expression of these 18 HERVs in HLA-A2 patients with basal breast cancer from TCGA cohort. HLA-A2–positive patients with a high or intermediate 18-HERV score had a significantly better overall survival than those with a low score (*P* = 0.0066) (Fig. 7C). This prognostic impact was not observed in the overall population (fig. S7A).

Last, we evaluated the antitumor activity of HERV-specific T cells against primary tumor cells by using organoids derived from the tumor of patient 8 (see fig. S7B and Materials and Methods). RNA-seq analysis confirmed the expression of the 18 epitope-containing CAHs at early and late passage (fig. S7C). Tumor organoids were cocultured with P1-, P6-, or CMV-specific CD8^+^ T cell clones in a 3D microscopy Nanolive experiment (E:T = 10:1). Whereas no activation of T cells was observed with CMV-specific T cells, P1- and P6-specific T cells exhibited signs of active proliferation associated with lysis of the organoids (Fig. 7D). Together, these last results suggest that HERV-specific T cells are induced during tumor development and may participate in the antitumor immune response.

## DISCUSSION

The first study providing a comprehensive characterization of the expression of HERVs in cancer was published in 2015 by Rooney *et al.* (*29*) using a small reference set of 66 HERVs. Recently, Smith *et al.* (*15*) developed the computational workflow HervQuant for the quantification of HERVs from RNA-seq data, based on Vargiu *et al.*’s (*19*) reference that compiles 3173 intact full-length HERV sequences. For our approach, these sequences underwent a first round of selection to retain HERVs specifically overexpressed in tumors. To limit the number of candidates for further validation, we next developed a machine learning–based approach using penalized regression, allowing a stringent selection by retaining only CAHs significantly associated with a CTL response.

This whole approach led to the selection of a limited number of cyt-HERVs per disease. We did not find a significant correlation with demethylation for all the HERVs studied. This may be due to the limitations of the methylation assay, which evaluates methylation in sites located at variable distances from the selected HERV locus. More recent techniques such as the assay for transposase-accessible chromatin with sequencing (ATAC-seq) could be used to more accurately address the epigenetic regulation of HERVs. The largest numbers of cyt-HERVs were in tumors that respond well to checkpoint inhibitors, such as not only in lung, head and neck, and renal cell carcinoma but also in colon cancer, regardless of the subtype and mismatch repair status. The immune impact of HERVs and cyt-HERVs in this latter disease remains to be addressed in a dedicated study to better understand the interactions and the mechanisms underlying the observed immune resistance in mismatch-repair–proficient colon cancer.

A limited number of shared T cell epitopes were identified by our approach. For practical reasons, we provided a proof of concept using HLA-A2, which is the most common human MHC class I molecule (*21*). It will be of interest to evaluate other HLA alleles and extend this pipeline of epitopes. A major finding of our study is the demonstration that HERV sequences are not only translated but also give rise to “virus-like” epitopes that are actually presented on HLA molecules on the surface of tumor cells. The quantity of peptide-HLA complexes per cell was low but sufficient to elicit a cytolytic T cell response, as previously reported (*30*, *31*). This suggests that the overexpression of epitope-containing HERVs in tumor cells would achieve the threshold of antigen detection by T cells expressing a TCR of high affinity, while healthy cells would express HERVs at undetectable levels. The fact that each epitope sequence is present in multiple HERVs located at different chromosomal loci may represent a major advantage compared to other tumor antigens by significantly decreasing the risk of selection of tumor clones losing the epitope.

The selected epitopes induce functional T cells with cytolytic properties, in agreement with the hypothesis that HERV-reactive T cells are not eliminated in the thymus during the negative selection process. Priming assays do not rule out the possibility that memory T cells are also activated. However, the low frequency of specific T cells, which could be estimated at less than 3 × 10^−6^ based on the analysis of specific TCRβ rearrangements in the T cell bulk before peptide stimulation, suggests that a low number of naïve T cells have been primed (*32*). The variations in responses observed between the donors may be due to the availability of the right TCR according to each individual T cell repertoire.

Our main assumption was that HERV-derived epitopes, due to their homology with virus sequences, may elicit high-affinity functional T cells. The selected epitopes induced CD8^+^ T cell clones of high predicted affinity and high functional avidity, as observed with virus antigens. The epitope specificity of these T cells is highlighted by the HLA-A2 restriction, the lack of cytotoxicity against nontumor HLA-A2–positive cells, the increased cytotoxicity observed with pulsed target cells, and structural modeling.

Last, we found preexisting HERV-specific T cells among TILs from TNBC samples, with variable frequencies and specificities among patients. This observation suggests that the selected epitopes (at least P1, P4, and P6) are efficiently processed and presented to T cells during tumor development. New methods are currently being developed to characterize the functional properties of these specific TILs in comparison with their peripheral counterparts. On the basis of the prognostic impact of epitope-containing HERVs in patients with HLA-A2, we will further assess the links between the expression levels of these HERVs, the frequency of HERV-specific T cells among TILs, and patient outcome.

Our results show HERV-derived targets as a class of virus-like tumor antigens shared by specific tumor subtypes. HERV-derived epitopes could be used in a strategy of innovative cancer vaccines, especially in tumors with low or intermediate mutational burden that are poor candidates for mutation-associated neoepitope-based vaccines. Furthermore, the characterization of TCRs specific to HERV-derived epitopes may also lead to the development of an immune cell therapy based on TCR-engineered T cells.

## MATERIALS AND METHODS

### Datasets

For RNA-seq data, raw fastq files were accessed from the National Center for Biotechnology Information Gene Expression Omnibus portal, under the accession number GSE58135 for Varley *et al.* (*25*) independent breast cancer dataset, GSE74246 for the sorted PBMC dataset (*33*), and GSE127825 and GSE127826 for the six mTEC samples (*34*). TCGA pancancer raw fastq files were accessed from the Genomic Data Commons portal (https://portal.gdc.cancer.gov/). Cell line data were accessed from the Broad Institute Cancer Cell Line Encyclopedia portal (https://portals.broadinstitute.org/ccle).

### HERV expression quantification

HERV expression was assessed using the HervQuant pipeline (*15*). Briefly, RNA-seq reads were mapped with STAR v2.7.3a (*35*) to the hg19 reference transcriptome compiled with the annotation of 3173 HERV sequences (*19*). Multimaps of ≤10 and mismatches of ≤7 were allowed, as in the original publication. BAM outputs were filtered for reads that mapped HERV sequences using SAMtools v1.4 (*36*) and then quantified using Salmon v0.7.2 (*37*). Raw counts were normalized to counts per million (CPM) total reads and then log_2_ + 1 transformed. Results were comparable to the original data published by Smith *et al.* (*15*).

### Quality check/sample filtering

Only primary solid tumor samples (TCGA code 01) were included, regrouping 9718 samples from 32 different cancer types, from which 9492 were analyzable for HERV expression. Quality check resulted in the complete removal of esophageal carcinoma and stomach adenocarcinoma samples because of a largely skewed HERV distribution, leading to the final analysis of 8893 samples from 29 different cancer types (fig. S1A and table S1).

### Immune signatures and genetic alterations

Phenotypic immune signatures were calculated with the Xcell method (*38*). For the TCGA pancancer samples, Xcell signatures were directly downloaded from the Xcell website (https://xcell.ucsf.edu/xCell_TCGA_RSEM.txt). For the GSM1401648 dataset, signatures were calculated for the whole dataset, and immune signatures were filtered after. IFN-γ signature was calculated by single-sample gene set variation analysis (*39*) based on the HALLMARK_INTERFERON_GAMMA_RESPONSE signature from the Molecular Signature Database (http://software.broadinstitute.org/gsea/msigdb/index.jsp). Enrichment scores were calculated for each sample per cancer type. The cytolytic activity (CYT_score) was calculated as the geometric mean of granzyme B (GZMB) and perforin (PRF1) expression, as previously described (*29*). TCGA pancancer genetic alterations were retrieved from Thorsson *et al.* (*40*).

### Cancer-associated and cyt-HERV annotation

To define cancer specificity, differential HERV expression was performed between tumor samples and their respective normal peritumoral matched tissues. Only TCGA studies with at least 10 peritumoral samples were included (*n* = 14 different cancer types). Differential HERV expression analysis was performed independently for each TCGA cancer type. Having filtered out any HERV expressed more than twofold in any normal tissue compared to its matched tumor, remaining HERVs overexpressed more than twofold in at least one cancer compared to its normal counterpart (peritumoral tissue) were considered cancer-associated.

To be annotated as potentially immunogenic, each CAH had to be associated with at least one phenotype (A) criterion and one functionality (B) criterion and not be overexpressed by T/NK cells (C). Phenotype criteria included an association with either CD4 or CD8^+^ T cell signatures as defined by the Xcell method (*38*). Function criteria included an association with either IFN-γ or the cytolytic activity, defined by the geometric mean of granzyme A (GZMA) and perforin (PRF1) expression (*29*). Normal PBMC expression was assessed in an independent dataset of sorted PBMCs from healthy donors (*33*). HERV expression was compared independently in T and NK cells to the rest of PBMCs.

### L1-penalized regression (Lasso)

Associations were calculated by Lasso regression using the glmnet and the c060 packages (*41*, *42*). Gaussian distribution was considered for the CYT score and the IFN-γ signatures, and Poisson distribution was considered for the Xcell signatures. HERVs were analyzed as log_2_(CPM + 1), requiring no further standardization. For each cancer type, a model was built on the basis of optimal parameters found with 10-fold cross validation. Each HERV with a positive coefficient in the final model (based on the lambda parameter minimizing the mean squared error) was considered to be associated with the variable.

### Epitope screening

ORF detection was performed using sixpack from EMBOSS v6.6.0.0 (*43*). Detected ORFs of more than 10 amino acids were then aligned to known HML-2 (HERV-K) Gag, Pro, Pol, Env, Rec, and Np9 proteins referenced in UniProt (*22*). Blast with optimal parameters for retrovirus was used (word size of 3, composition-based statistics, no “low-complexity region” filter). Conserved sequences aligned with Gag and Pol proteins with more than 90% identity and an *e* value of <0.05 were then screened for predicted HLA-A*02 strong binders using MHCflurry v1.3 (*44*). Peptides with a rank of ≤0.5 percentile were considered to be strong binders. The human proteome was downloaded on UniProt (ID: UP000005640) to validate the absence of match before peptide synthesis and in vitro validation.

### Cumulative expression score

The π value score was defined for each HERV and each tissue comparison (TNBC versus peritumoral tissue) as the product of the log_2_ fold change of expression and the log_10_ of the inverse *P* value, according to the method proposed by Xiao *et al.* (*26*). The cumulative expression score was calculated by summing the π values of all the HERVs containing the epitope sequence (including CAHs and other HERVs).

### Analysis of peptidome proteomic datasets

Raw MS/MS datasets were downloaded from CPTAC (*23*) for breast cancer studies (*45*, *46*). Retrieved MS/MS spectra were converted to MGF format using msconvert from proteowizard (*47*). Then, the list of peptides was analyzed using the standalone version of Pepquery (v.1.6.2.0) (*48*). The used command line was as follows: java -Xmx10G -jar pepquery.jar -fixMod 6,62,108 -varMod 117 -maxVar 3 -c 1 -tol 10 -tolu ppm -minScore 12 -e 1 -um -hc TRUE -n 1000 -itol 0.05 -m 1 cpu 12 -pep ${peptides_list} -db ${Reference_database} -ms ${MS_database} -o ${output_directory}. For the second dataset, the fixmod and varMod in the command line were adapted similar to the following: -fixmod 6,103,157 -varMod 101,117.

### Riboseq analysis

Ribosome profiling data were retrieved from a previously published study (*27*). Raw fastq files were preprocessed as described in the initial publication. Briefly, adapter sequences were trimmed from raw data using cutadapt 1.1 with parameters (--quality-base=33 -O 12 -m 20 -q 5) and mapped to our reference hg19-HERV transcriptome.

### Statistical analysis

All analyses were performed using R statistical software version 3.6.0. Differential HERV expression analysis was performed using DESEQ2 v1.24.0 (*49*), and logarithmic fold changes were shrunk with the apeglm package (*50*).

### Biological samples

Blood from healthy donors was obtained from the “Etablissement Français du Sang” (Lyon). Fresh TNBC samples (*n* = 11) were provided by the tissue bank of Centre Léon Bérard (CLB) (BB-0033-00050, CRB-CLB, Lyon, France; French agreement number AC-2013-1871), after approval from the Institutional Review Board and ethics committee (L-06-36 and L-11-26) and patient written informed consent, in accordance with the Declaration of Helsinki.

### Peptide synthesis

Peptides were synthetized at JPT Peptide Technologies (GE, EU) with a specification and a purity of >90%. Lyophylized powder was resuspended in 1% dimethyl sulfoxide (DMSO) distilled water.

### Cell lines

MDA-MB-231 basal breast cancer epithelial cells were obtained from the American Type Culture Collection (ATCC catalog name HTB-26) and cultured in 10% fetal bovine serum (FBS), Dulbecco’s modified Eagle’s medium (DMEM) (Gibco, FR, EU), 1% penicillin/streptomycin, and 1% l-glutamine. HMEC primary cells were obtained from PromoCell (GE, EU) and cultured in mammary epithelial growth medium (PromoCell, GE, EU).

### In vitro priming assays

PBMCs were obtained by Ficoll density gradient centrifugation (Eurobio, FR, EU). They were rapidly thawed at 37°C, extensively washed, and kept at room temperature or overnight at 37°C before assessing their viability. PBMCs (0.15 × 10^6^) per well were cultured in 96-well plates with AIM V medium (Gibco, FR, EU) enriched with R-848 (5 μg/ml; resquimod), high–molecular weight poly-IC (polyinosine-polycytidylic acid) (10 μg/ml; both Invivogen, FR, EU), interleukin-2 (20 IU/ml; IL-2; PROLEUKIN aldesleukin, Novartis Pharma, CH, EU), and the peptide of interest (10 μg/ml) at day 0. After 3, 6, and 10 days, 100 μl of medium was replaced by enriched fresh medium (IL-2 and peptide only at day 6 and IL-2 only at day 10) and splitted if necessary. On day 12, cells were collected and counted for analysis.

### Feeding protocol

Dextramer single-cell–sorted CD8^+^ T cells were expanded on a feeder composed by 35-gray irradiated allogeneic PBMCs and B lymphoblastoic cell lines in a ratio of 10:1. Feeder cells were plated in a 96-well round-bottom plate at a concentration of 0.10 × 10^6^ cells per well in RPMI 5% human serum with phytohemagglutinin-L (1.5 μg/ml; Merck KgAa, GE, EU) and IL-2 (150 IU/ml; Novartis Pharma, CH, EU), and up to 5 × 10^3^ sorted cells were added per well. Cells were cultured for 14 days, and medium was replaced when needed with fresh IL-2–enriched RPMI 5% human serum. This process was repeated if needed.

### TCR immunosequencing

DNA from specific CD8^+^ T cells and the corresponding bulk PBMCs was extracted using the QIAGEN QIAmp DNA Blood Micro Kit (QIAGEN, GE, EU) and sent for TCR survey and deep analysis to Adaptive Biotechnologies (WA, USA).

### Generation and refinement of 3D models

The full TCR sequences of both α and β chains were reconstructed for each T cell clone from the results of the immunosequencing as previously published (*51*). For variable domains, TRA and TRB complementarity-determining region 3 (CDR3) nucleotide sequences were obtained from immunosequencing (Fig. 5A) and the 5′ and 3′ ends of the TRAV and TRBV regions were obtained from the International Immunogenetics Information System online database. Human constant domains of TRA and TRB were added in 3′ of the variable domains to reconstitute the full-length TCR. These full-length TCR sequences, together with the MHC and peptide sequences, were submitted to the CBS TCRpMHCmodels-1.0 web server, specifically developed for the automatic structural modeling of TCR-pMHC complexes (*52*) using template-based modeling. TCR residues are renumbered using a standardized procedure (*53*, *54*). The initial models generated by the web server were further refined in four rounds using a protocol adapted from Bobisse *et al.* (*28*). Briefly, the CDR loops were refined by pairs using Modeller software version 9.25 (*55*, *56*): CDR loops α1/α2 at round 1, α1/α3 at round 2, β1/β2 at round 3, and β1/β3 at round 4. Only the residues in coil conformations in the initial models were refined. In each round, 500 models were generated, and the best model based on the Modeller internal DOPE score was selected and used as an input for the next round.

At the end of the four rounds, a representative model was chosen on the basis of the consensus of unweighted contacts as follows: Contacts between residues at the TCR-pMHC interface (defined by a distance lower than 5 Å between heavy atoms) were counted in the set of 4*25 models with best DOPE scores of each refinement round. Then, among the 25 models with best DOPE scores in the final round, the model with the highest number of recurrent contacts [referred as un-normalized CONSRANK score (*57*, *58*)] is elected as the representative model.

A quantitative view of potential stabilizing interactions between TCR and pMHC is provided by the frequencies of interresidue contacts observed in the set of 4*25 models with best DOPE scores of each refinement round (fig. S5D). These TCR-pMHC interactions concur with previous studies (*59*). Whereas CDR1 and CDR3 loops of both TCRα and TCRβ chains interact with both the MHC and the peptide, the CDR2 loops interact mostly with the MHC molecule.

#### *3D structure analysis*

The structural similarity between representative models of different complexes was assessed by the root mean square deviation between backbone CDR loops computed with UCSF Chimera (fig. S5, B and C) (*60*). As expected, structural variability was highest within CDRβ3 loops. UCSF Chimera was also used for hydrogen bonds and hydrophobic contact detection and structure visualization.

#### *Binding affinity prediction*

The binding affinity was predicted using the prodigy method (*61*), which uses a linear model based on the number and types of contacts at the interface. For each complex, instead of running one prediction on the representative model, we averaged the predictions obtained for the 25 models with the best DOPE scores obtained at round 4 of the refinement protocol.

### Fluorospot

After coculture of PBMCs or CD8^+^ T cells with T2 cells pulsed or not with the peptide in a ratio of 10:1 in AIM V medium (Gibco, FR, EU) at 37°C and 9% CO_2_ for 24 hours, double-color Fluorospot (CTL GmbH, CA, USA) with IFN-γ AF488 and Grz-b CTL-red was performed according to the manufacturer’s instructions. Revelation plate was read on an ImmunoSpot S6 ULTIMATE UV image analyzer and analyzed with the ImmunoSpot analysis software.

### Functional avidity

Dextramer-isolated specific CD8^+^ T cells for the selected peptides (P1, P2, and P6) were used in a functional avidity IFN-γ production test by enzyme-linked immunosorbent assay (IFN-γ ELISA, Thermo Fisher Scientific, FR, EU). Two CMV T cell clones specific for the immuno-dominant epitope N9V (NLVPMVATV) were used. N9V-1 corresponds to phosphoprotein 65 (pp65) dextramer-selected CD8^+^ T cells, and N9V-2 is a CD8^+^ T cell clone provided by H. Vie. Functional avidity of specific CD8^+^ T cell responses was assessed by performing limiting peptide dilutions from 10^−4^ to 10^−9^ M (log) charged on T2 cells pulsed for 5 hours. After wash, peptide-pulsed T2 cells were cocultured with specific CD8^+^ T cell in a ratio of 1:1 in AIM-V medium (Gibco, FR, EU) supplemented with 5% human serum. After 18 hours, supernatants were collected, and ELISA was performed. The peptide concentration required to achieve a half-maximal cytokine response (EC_50_) was determined (GraphPad Prism, version 6.0 for Windows was used for EC_50_ value determinations, *R* > 0.98).

### Epitope validation and quantification by MS

Epitope validation and quantification by MS were performed by Complete Omics Inc. (MD, USA) according to the method previously described (*62*) with further modifications. In brief, a total of 300 million cells were lysed, and peptide-HLA complexes were immunoprecipitated using self-packed Valid-NEO neoantigen enrichment column preloaded with anti-human HLA-A, B, and C antibody clone W6/32 (BioXCell). After elution, dissociation, filtration, and cleanup, peptides were lyophilized before further analysis. Transition parameters for each epitope peptide were examined and curated through Valid-NEO method builder bioinformatic pipeline to exclude ions with excessive noise due to coelution with impurities and to boost up the detectability through recursive optimizations of significant ions. Absolute copy numbers of peptides presented on the cell surface were calculated on the basis of the quantification using the heavy isotope–labeled peptides. The MS data have been deposited via ProteomeXchange and can be accessed through identifier PASS01698.

### Live imaging

Cells were plated in DMEM (Gibco, FR, EU) medium, 10% fetal bovine serum, and 1% penicillin/streptomycin. For IncuCyte analysis, medium was removed from the 96-well plate after overnight cell adhesion. A blocking HLA-A2 antibody (GeneTex, clone BB7.2, GTX75806; CA, USA) was added in AIM-V medium (Gibco, FR, EU) for 1 hour, according to conditions. T cells were then added in an E:T ratio of 2:1 in the presence of IncuCyte Cytotox dye (Essen Bioscience, UK, EU) for cell death quantification. A 48-hour live imaging was performed at 37°C and 5% CO_2_ with IncuCyte Zoom. Cell death was calculated as the total number of counted stained cells corrected by the number of counted stained cells at baseline. Maximum killing was established using DMSO. Specific lysis was calculated according to the following formula: % specific lysis = (((HERV-specific T cells induced target cell death − spontaneous target cell death) − (nonspecific dextramer-negative T cell–induced target cell death − spontaneous target cell death))/(DMSO-induced target cell death − spontaneous target cell death)) × 100. For Nanolive imaging, T cells were then added with an E:T 10:1, and phase imaging was performed every minute using Nanolive microscope 3D cell explorer.

### Tumor dilacerations: Organoids and TIL expansion

Tumor tissues were dissected into fragments of approximately 1 mm^3^ and dilacerated with collagenase IV and deoxyribonuclease for 45 min in 20% SVF-enriched RPMI. The tumor lysate was centrifuged at 1500 rpm for 5 min and resuspended in 5% human serum–enriched RPMI. Cells were counted and plated at a density of 5 × 10^4^ cells per well in a flat-bottom 96-well plate with anti-CD3 anti-CD28 Dynabeads (Dynabeads, Gibco, EU) and IL-2 at 100 IU/ml in a bead-to-cell ratio of 1:4.

For organoids, a part of the tumor lysate (3 million to 10 million of cells) was resuspended in 10 ml of Advanced DMEM/F12 medium. Cells were centrifuged at 500 rcf for 10 s and then resuspended in full medium. This protocol was repeated three to five times according to the cell number at the beginning to enrich the cell suspension in epithelial cells. These cells were then cultured according to the protocol previously described by Driehuis *et al.* (*63*).

### Multiparametric flow cytometry

T cells were counted and cocultured with T2 cells loaded or not with the cognate peptide in a 5:1 ratio. After 1 hour, CD107a antibody (BD, clone H4A3) was added in each well with Golgi plug (1:1000) (10 μg/ml; BD, FR, EU). After 5 hours, viability and surface and intracellular staining procedures were performed. To assess cytokine expression in CD8^+^ T cells, an intracellular staining with the FoxP3 fixation and permeabilization kit (Thermo Fisher Scientific, Life Technologies, CA, USA) was performed according to the manufacturer’s instructions.

Dextramer staining was performed on PBMCs after a 12-day culture (priming protocol) or on TILs expanded for 14 days after tumor dilaceration. Cells were washed in 2 ml of washing buffer [phosphate-buffered saline + 2% FBS + 2 mM EDTA (Sigma-Aldrich, MI, USA)] and stained for 10 min with dextramers (Immudex ApS, DK, EU) at room temperature before viability and surface marker staining. Washing was performed two times to avoid nonspecific dextramer staining. CMV pp65 NLVPMVATV was used as a positive control. For TIL analysis, a dextramer complexed to a non-natural irrelevant peptide (ALIAPVHAV) was used as a negative control.

All samples were analyzed on an LSRFortessa (BD Biosciences, FR, EU) with conserved settings throughout the entire study. Data were analyzed using FlowJo software (Tree Star v10.4, NJ, USA).

## Notes

View/request a protocol for this paper from *Bio-protocol*.
