# Supplementary material for: Identification of shared tumor epitopes from endogenous retroviruses inducing high-avidity cytotoxic T cells for cancer immunotherapy
Source: Sci Adv. 2022 Jan 26;8(4):eabj3671. doi: 10.1126/sciadv.abj3671 (PMC8791462; doi:10.1126/sciadv.abj3671)
Supplement: Supplementary file 1 — Supplementary Methods Figs. S1 to S7 Tables S1 to S4 Legends for movies S1 to S5 [file sciadv.abj3671_sm.pdf]

Science Advances
AAAS

Supplementary Materials for

Identification of shared tumor epitopes from endogenous retroviruses inducing high-avidity cytotoxic T cells for cancer immunotherapy

Paola Bonaventura, Vincent Alcazer, Virginie Mutez, Laurie Tonon, Juliette Martin, Nicolas Chuvin, Emilie Michel, Rasha E. Boulos, Yann Estornes, Jenny Valladeau-Guilemond, Alain Viari, Qing Wang, Christophe Caux, Stéphane Depil*

*Corresponding author. Email: stephane.depil@lyon.unicancer.fr

Published 26 January 2022, *Sci. Adv.* **8**, eabj3671 (2022)
DOI: 10.1126/sciadv.abj3671

The PDF file includes:

Supplementary Methods
Figs. S1 to S7
Tables S1 to S4
Legends for movies S1 to S5

Other Supplementary Material for this manuscript includes the following:

Movies S1 to S5

SUPPLEMENTARY METHODS

Methylation analysis

Pre-processed TCGA pancancer Beta-value from Illumina 450K methylation data were accessed from the GDC portal from the PanCanAtlas description page. Riboseq raw fastq files were accessed from the GEO portal under the accession number GSE69923. Samples under glutamine starvation conditions were omitted from the analysis.

For each cyt-HERV, a local methylation status was calculated according to the mean beta-value of the 10 nearest surrounding probes. Spearman's correlations were then calculated independently for each cyt-HERV. HERVs with a Spearman's $R < -0.3$ and an adjusted $p < 0.05$ were considered to be associated with a local demethylation status, and HERVs with a Spearman's $R > 0.3$ and an adjusted $p < 0.05$ with a local methylation status.

Binding affinity analysis

The 6 selected and control peptides were synthetized to perform a high throughput quantitative binding assay to MHC allele HLA-A2. ProImmune's Class I REVEAL® Rapid Epitope Discovery System detection is based on the presence or absence of the native conformation of the MHC-peptide complex. Each peptide is given a score relative to the positive control peptide, which is a known T cell epitope. The score is reported as a percentage of the signal generated by the test peptide versus the positive control peptide. REVEAL® on- and off-rate assays give a quantitative picture of the binding properties of individual peptides. On and off-rates for peptides that have passed the MHC-binding assay are measured at six points over 48 hours (on-rate) and 24 hours (off-rate). Results are presented as half-life values. Peptides are given a kinetic score based on the results of both rates; a higher score indicates a better candidate T cell epitope. Finally the 'R' score is defined for each peptide. This incorporates the kinetic score with the MHC-peptide binding assay score; the higher the R score, the better the epitope.

Dendritic cell-based priming assays

29 to 34-mer synthetic long peptides corresponding to the native Gag or Pol polypeptide sequence and containing P1, P2, P4 or P6 epitopes were synthetized by JPT peptide Technologies (GE, EU).

Monocytes were isolated from PBMCs by positive selection of $CD14^{+}$ cells (Myltenyi, GE, EU). The negative fraction was considered as peripheral blood lymphocytes (PBLs). Cells were frozen

in FBS 10% DMSO and kept at -80°C. Monocyte-derived dendritic cells (MoDCs) were generated from 6-day cultures of $CD14^+$ monocytes in complete RPMI medium supplemented with 10 % FCS and recombinant human GM-CSF (100 ng/mL) and IL-4 (50 ng/mL). MoDCs were pulsed either with short 9-mer peptides (10µg/mL) overnight with 10 ng/ml of LPS or with long peptides (1µM) with TNF-α (20µg/ml) plus Poly-IC (40µg/ml) and washed before co-culture with isolated PBLs for 6 days (MoDCs:T-cells ratio 1:10) in 96 round wells plate. After 6 days T cells were counted and restimulated with autologous MoDCs for 6 more days. n=5 donors were tested for each short peptide and n=4 donors were tested for each long peptide.

HCC1599 cell line and Nanolive imaging

The non-adherent TNBC cell line HCC-1599 (ATCC number: CRL-2331) growing in multicellular aggregates was cultured in RPMI-1640 supplemented with 10% SVF and 1% P/S in 6 well plates for cell expansion.

For Nanolive imaging, cells were suspended to single cells and plated on a pre-coated (PBS+2% BME) µ-dish 24h before imaging. T cells were added directly before the 10-hour imaging.

Supplementary figure 1

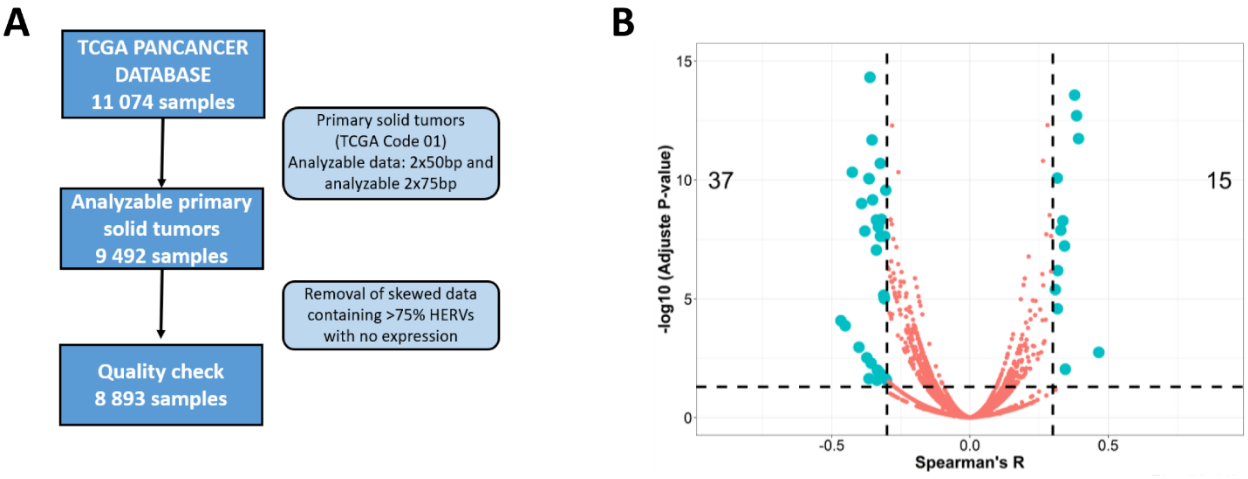

Supplementary Fig. S1. TCGA pancancer flow-chart and methylation analysis

(A) Flow-chart of included samples for the pancancer study. Only primary solid tumors samples were included. Studies with insufficient data quality were excluded, leading to the final analysis of 8,893 primary tumor samples from 29 different cancer types.

(B) Correlation between cyt-HERVs expression and local methylation state in TCGA pancancer dataset. For each HERV, the mean beta-coefficient of the 10 nearest methylation probes was calculated. Spearman's correlation between each individual HERV in each cancer type and its median surrounding beta-coefficient is shown.

Cyt-HERVs: HERVs associated with cytotoxic response, TCGA: The Cancer Genome Atlas

Supplementary figure 2

A

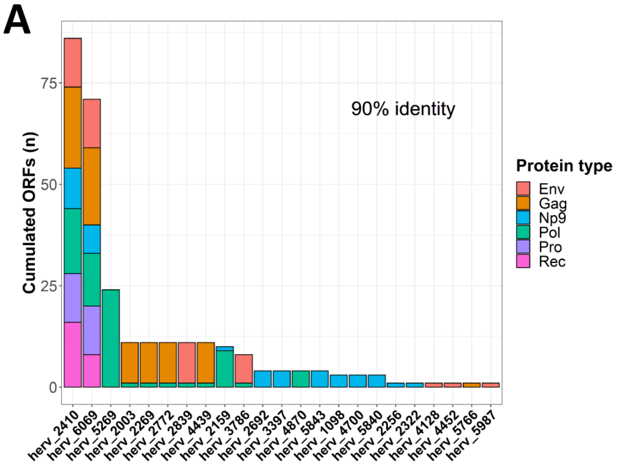

B

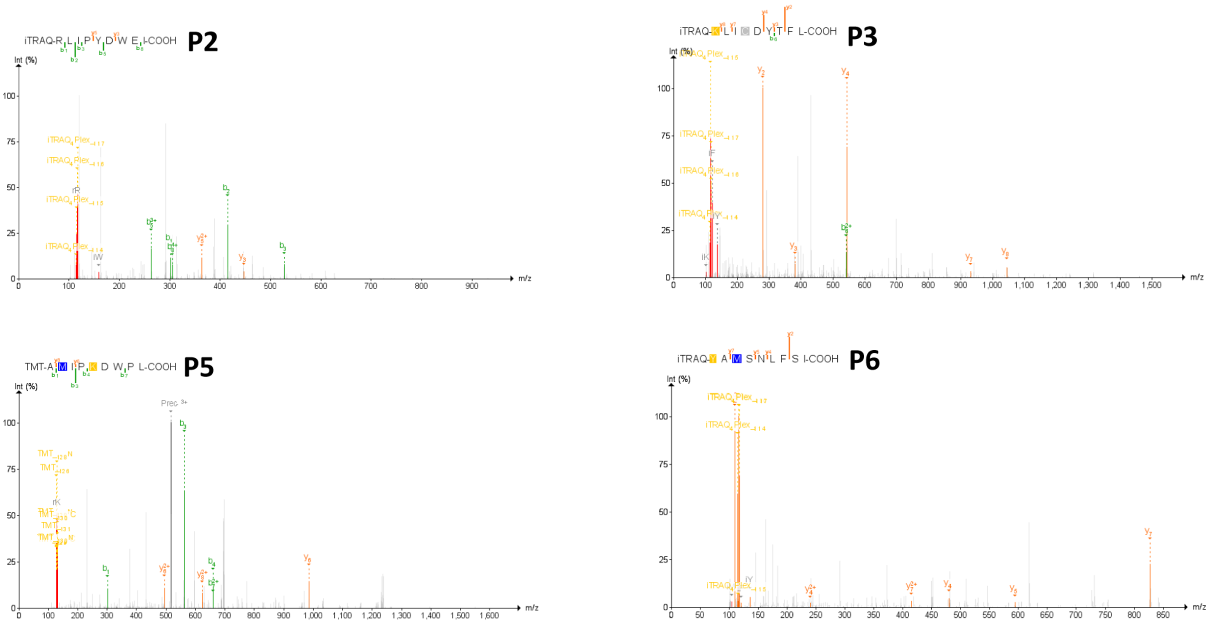

Supplementary Fig. S2. Open reading frames identification and peptides mass spectra

(A) Top HERVs with the highest number of conserved ORFs from the HML-2 family among cyt-HERVs. ORFs with at least 90% identity with known HML-2 proteins are represented.

(B) MS/MS detection of P2, P3, P5 and P6 epitopes in samples from the CPTAC or TCGA breast cancer prospective dataset. MS/MS spectrum is identified by Pepquery analysis (Peptide Spectrum Match, p values 0.0010, 0.0010, 0.0010 and 0. 0031, respectively)

Supplementary figure 3

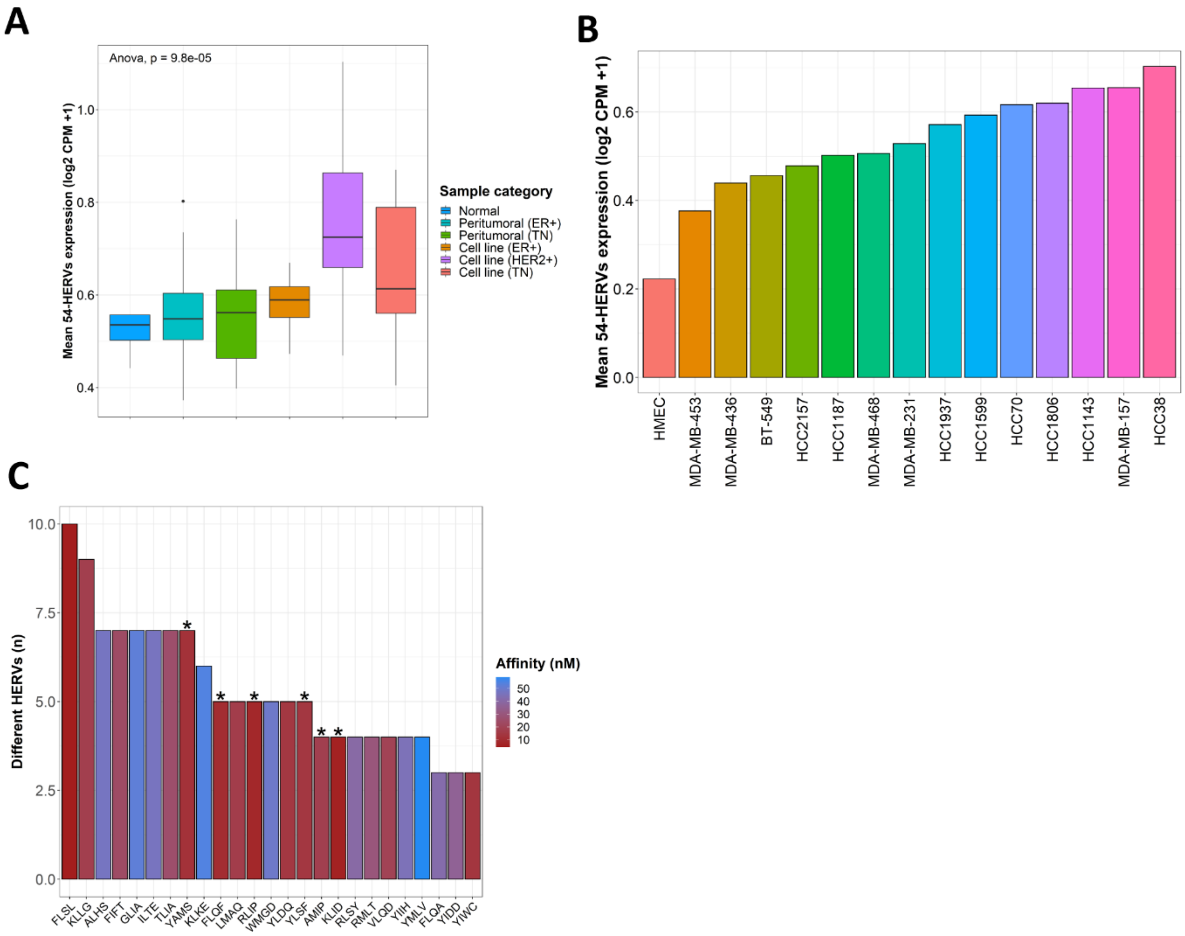

Supplementary Fig. S3. TCGA basal breast cancer cyt-HERVs and peptide analysis

(A) Mean expression of the 54 cyt-HERVs from TCGA basal subtypes is represented in cell line samples from Varley's independent dataset.

(B) Mean expression of the 54 cyt-HERVs from TCGA basal subtypes in TNBC cell line samples from the CCLE database.

(C) Number of different HERVs containing the top 25 peptides. Affinity is colored on a red-blue scale.

Cyt-HERVs: HERVs associated with cytotoxic response, ORFs: Open Reading Frames, TCGA: The Cancer Genome Atlas, TNBC: Triple-Negative Breast Cancer

Supplementary figure 4

A

Peptide ID	Sequence	REVEAL® score	On-rate T1/2 (h)	Off-rate T1/2 (h)	Kinetic score	R-score
1	FLQFKTWWI	88.70	8.35	12.22	1.46	1.30
2	RLIPYDWEI	45.11	5.78	20.49	3.55	1.60
3	KLIDCYTFL	80.30	11.39	40.61	3.57	2.86
4	YLSFIKILL	58.96	13.18	20.76	1.58	0.93
5	AMIPKDWPL	77.53	7.59	35.08	4.62	3.58
6	YAMSNLFSI	55.28	5.42	46.95	8.67	4.79
**Positive control**	**-**	**100.00**	**11.83 +/- 3.00**	**85.58 +/- 34.42**	**7.21 +/- 0.48**	**7.24**

B

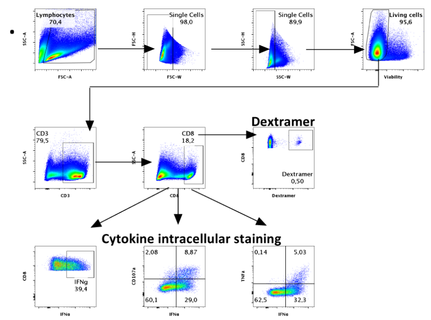

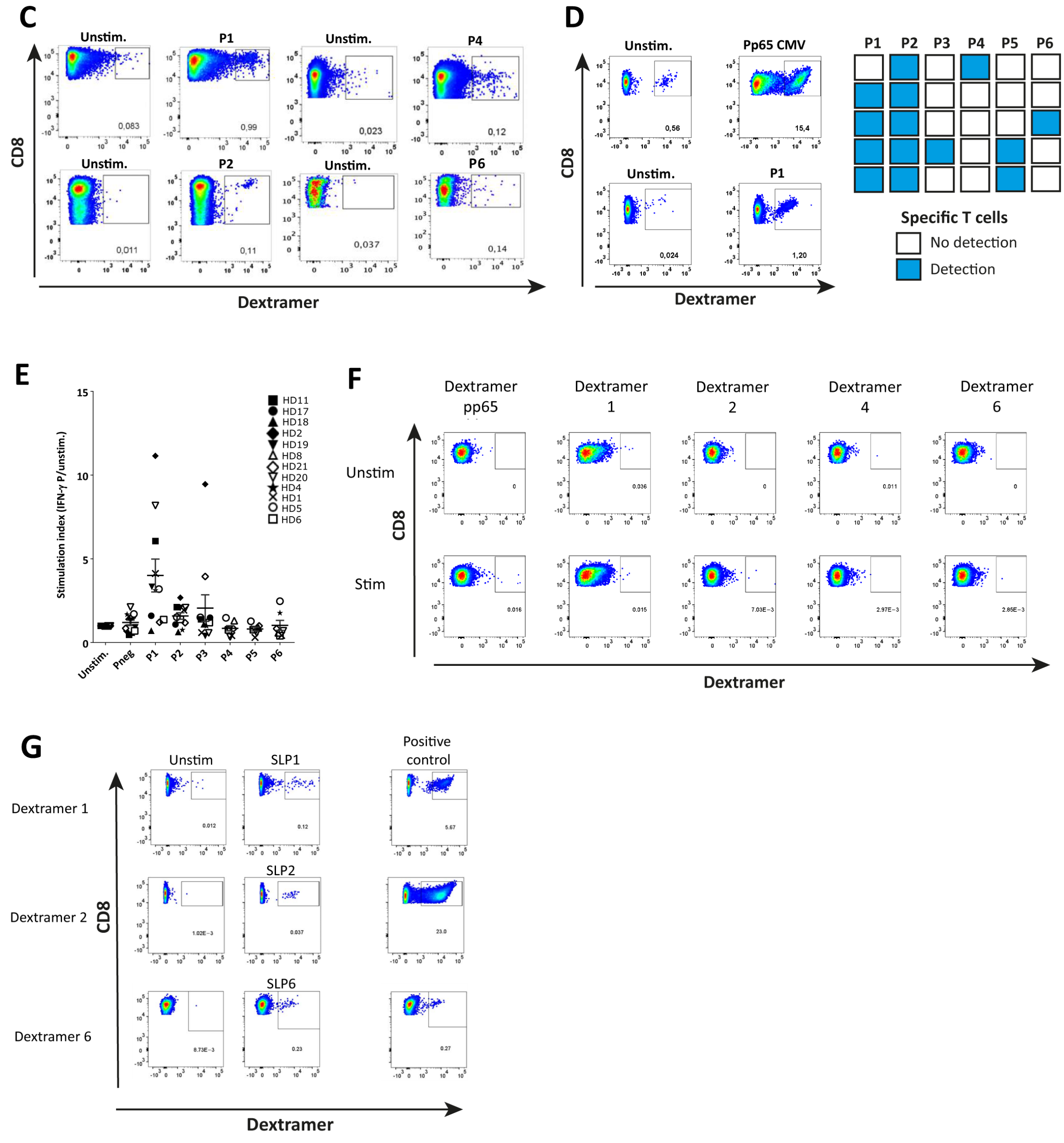

Supplementary Fig. S4. HERV-derived epitopes CD8$^+$ T cell responses and gating strategies

(A) Summary table of the binding rate data and kinetic scores for the 6 peptides. Reveal scores ≥ 45% indicate good binders (see supplementary methods for the definitions).

(B) Gating strategy for the analysis of flow cytometry data for dextramer staining protocol and polychromatic intracellular cytokine (IFN-γ and TNF-α) and CD107a CD8$^+$ T cells. Gates on lymphocytes morphology, single cells in SSC and FSC, CD3$^+$ T cells, CD8$^+$ T cells to identify double CD8$^+$dextramer+ cells. The same gating strategy was used to identify IFN-γ+ TNF-α+ CD107a+ CD8$^+$ T cells.

(C) Representative panels of dextramer staining in unstimulated (left panel for each peptide) and peptide-stimulated (right panel for each peptide) CD8$^+$ T cells for different peptides (P1, P2, P4, P6) after stimulation with peptides and TLR ligands using healthy donors PBMCs

(D) Representative panels of dextramer staining in unstimulated (left panels) and peptide-stimulated (right panels) CD8$^+$ T cells using monocyte-derived dendritic cells. Right: summary table of the results obtained in 5 HLA-A2-positive healthy donors (HD12-16).

(E) Summary (individual values and median) of IFN-γ stimulation index in 12 healthy donors PBMCs after stimulation with peptides and TLR ligands. IFN-γ stimulation index is defined as the ratio between specific IFN-γ+CD8+ T cells (peptide stimulated) and unspecific IFN-γ+CD8+ T cells (non-stimulated with the peptide). Experiments performed independently (using different PBMC aliquots) of the dextramer staining for HD1, HD2, HD4, HD5, HD6 and HD8.

(F) Representative panels (n=5) of dextramer staining in unstimulated (upper panels) versus stimulated (lower panels) PBMCs from an HLA-A2-negative healthy donor

(G) Representative panels of dextramer staining in unstimulated (left panels), long peptide-stimulated (central panels) and MART-1 stimulated (right panels, positive control with staining with the corresponding dextramer) CD8$^+$ T cells using monocyte-derived dendritic cells (n=4). Line 1: T cells stimulated (central panel) or not (left panel) with a P1-containing SLP (SLP1) (HD24); line 2: T cells stimulated (central panel) or not (left panels) with a P2-

containing SLP (SLP2) (HD25); line 3: T cells stimulated (central panel) or not (left panel) with a P6-containing SLP (SLP6) (HD26).

IFN: Interferon, PBMCs: Peripheral Blood Mononuclear Cells, TLR: Toll-Like Receptor, TNF: Tumor Necrosis Factor; unstim: unstimulated; stim: stimulated; SLP: synthetic long peptide.

Supplementary figure 5

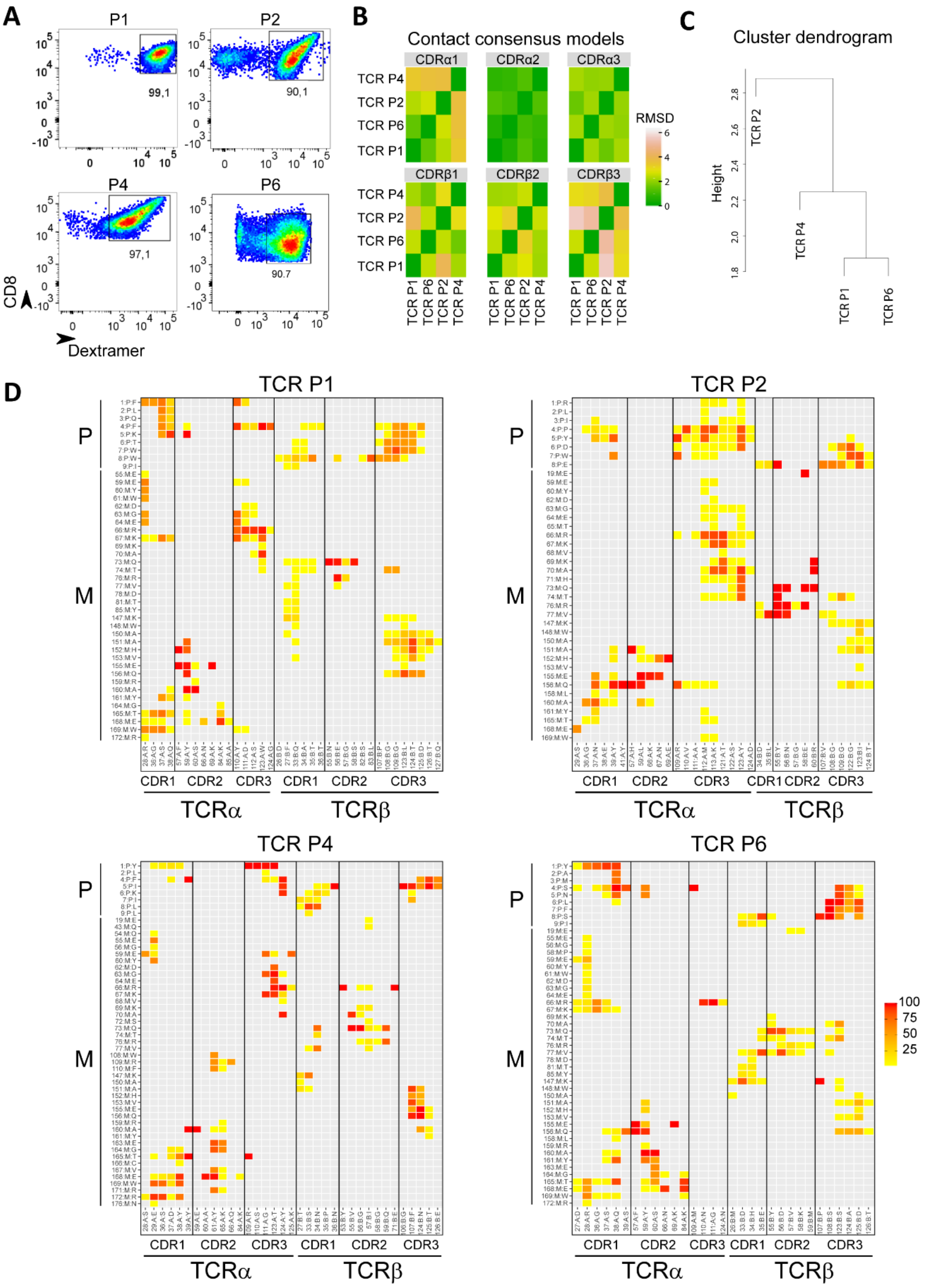

Supplementary Fig. S5. Dextramer staining after feeder expansion and analysis of 3D structural models

(A) Representative plots of dextramer staining of specific $CD8^+$ T cells after feeder expansion. Dextramer staining was between 90 and 99% for TCR sequencing.

(B) RMSD matrix between CDR loop conformations on the refined representative models. The RMSD is averaged over the 6 CDR regions with equal weights.

(**C**): Clustering tree obtained from the RMSD matrix in B. High structural similarity is observed between TCR P1 and TCR P6 CDR loops, which share the same TCR alpha sequences. On the contrary, TCR P4 and TCR P2 CDR loops are structurally more distant.

(D) Frequencies of inter-residue contacts obtained from the subsets of 25*4 models generated during the refinement protocol, that were used to identify the representative models.

Supplementary figure 6

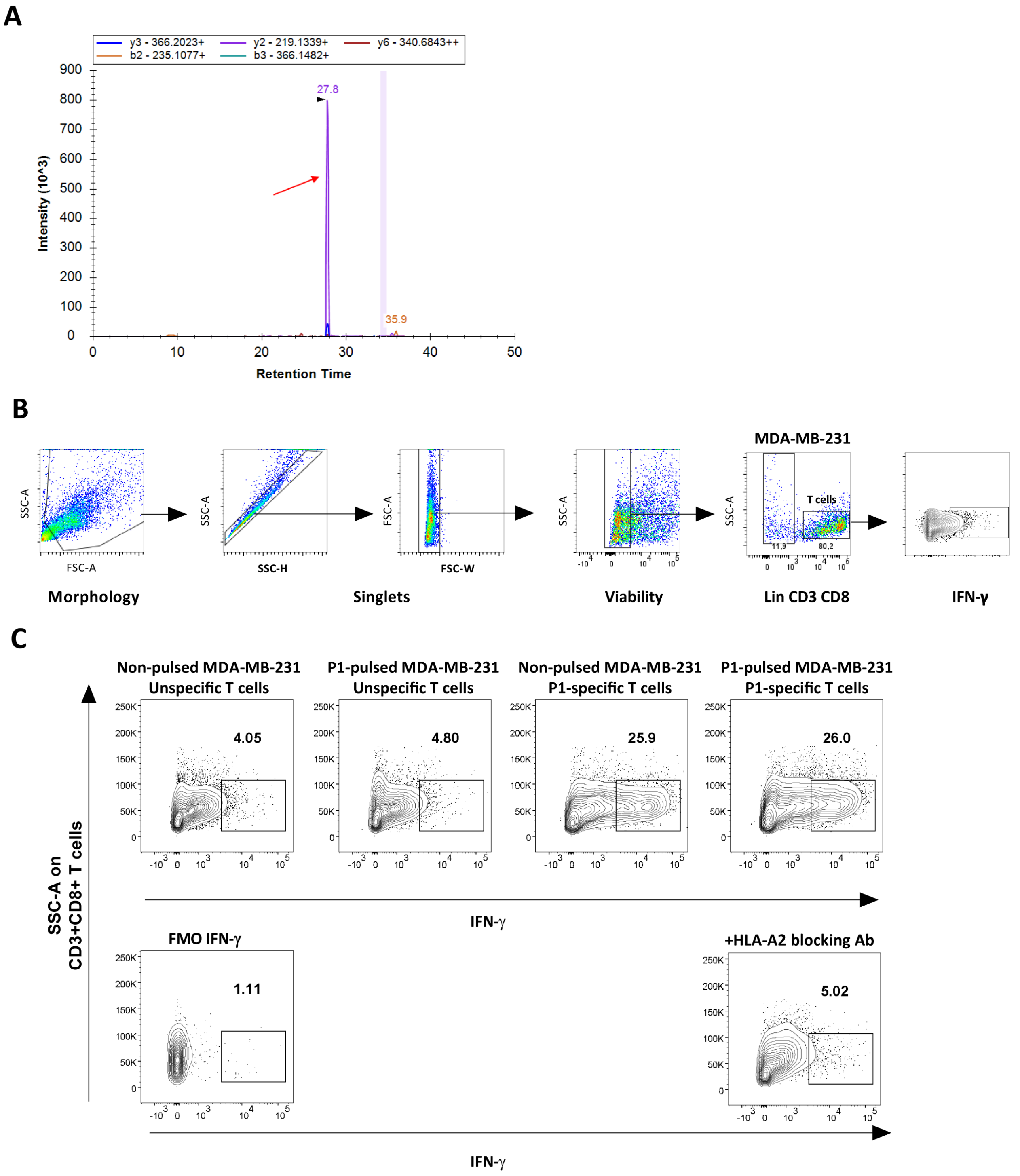

Supplementary Fig. S6. Epitope validation by MS and flow cytometry assays on T cells co-cultured with tumor cells

(A) Valid-NEO Transitions of peptide P6 (YAMSNLFSI) are shown on the chromatogram.

(B) Gating strategy and representative plots for FACS analysis of IFN- γ produced by T cell co-cultured with MDA-MB-231

(C) Representative plots of IFN-γ analysis of $CD8^+$ specific T cells co-cultured with MDA-MB-231 in the different culture conditions.

Supplementary figure 7

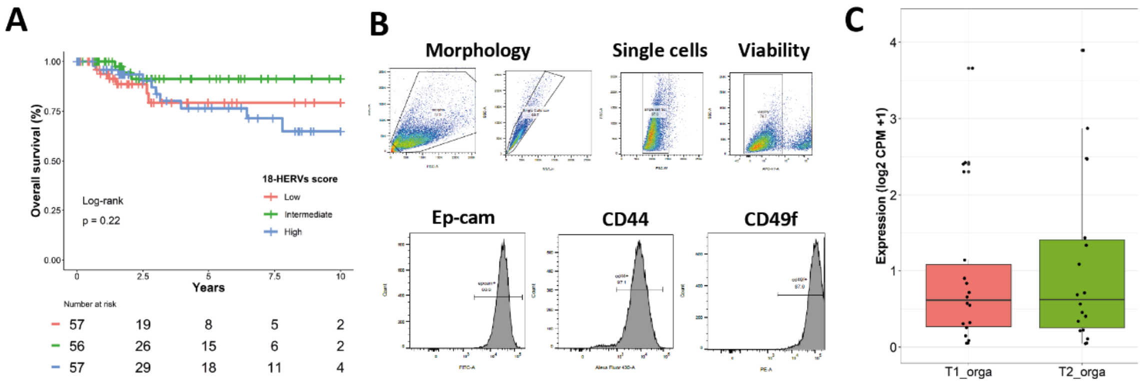

Supplementary Fig. S7. HERV expression in TNBC patients and organoids cell surface markers

(A) Overall survival according to 18-HERVs score in TCGA TNBC patients (all HLA subtypes, n=170). Patients were divided in three groups: blue line, high expression (n=57); green line, intermediate expression (n=56); red line, low expression (n=57)

(B) Flow cytometry analysis of organoid-constituting cells including Ep-cam, CD44 and cD49f epithelial markers

(C) Boxplot of mean expression of the 18 Cyt-HERVs expression in organoids RNAseq at early (T1) or late (T2) culture passage. Mean expression is represented in log2 CPM +1.

HLA: Human Leukocyte Antigen, TCGA: The Cancer Genome Atlas, TNBC: Triple-Negative Breast Cancer

SUPPLEMENTARY TABLES

Tumoral samples	
**TCGA Study**	**Samples (n)**
BRCA	1092
UCEC	545
KIRC	533
HNSC	520
LGG	516
LUAD	515
THCA	505
LUSC	501
PRAD	497
COAD	457
BLCA	407
LIHC	371
CESC	304
KIRP	290
SARC	259
PCPG	179
PAAD	178
READ	166
GBM	154
TGCT	150
THYM	120
SKCM	103
MESO	87
UVM	80
ACC	79
OV	78
KICH	66
UCS	57
DLBC	48
CHOL	36
TOTAL	8893

Peritumoral samples	
**TCGA Study**	**Samples (n)**
BRCA	113
KIRC	72
LUAD	59
THCA	59
PRAD	52
LUSC	51
LIHC	50
HNSC	44
COAD	41
UCEC	35
KIRP	32
KICH	25
BLCA	19
READ	10
TOTAL	662

Table S1: Number of samples from each cancer type included in the study.

LAML, ESCA and STAD have been completely removed from the study due to skewed distribution. For normal tissue comparison, only studies with at least 10 peritumoral samples have been included.

LAML: Acute Myeloid Leukemia, ESCA: Esophageal Carcinoma, STAD: Stomach Adenocarcinoma

Query_ID	Subject_ID	P_ident	Align_len	Mismatc	Gap_ope	Q_start	Q_end	S_start	S_end	E_value	Bit_scor
Peptide_1	sp|Q7LDI9|GAK6_HUMAN	100.000	9	0	0	1	9	363	371	0.050	27.7
Peptide_1	sp|P62685|GAK8_HUMAN	100.000	9	0	0	1	9	363	371	0.050	27.7
Peptide_1	sp|Q9YNA8|GAK19_HUMAN	100.000	9	0	0	1	9	363	371	0.050	27.7
Peptide_1	sp|P62683|GAK21_HUMAN	100.000	9	0	0	1	9	363	371	0.050	27.7
Peptide_1	sp|P63130|GAK7_HUMAN	100.000	9	0	0	1	9	363	371	0.051	27.7
Peptide_1	sp|P63126|GAK9_HUMAN	100.000	9	0	0	1	9	363	371	0.051	27.7
Peptide_1	sp|P63145|GAK24_HUMAN	100.000	9	0	0	1	9	363	371	0.051	27.7
Peptide_1	sp|P62684|GA113_HUMAN	100.000	9	0	0	1	9	363	371	0.051	27.7
Peptide_1	sp|P87889|GAK10_HUMAN	100.000	9	0	0	1	9	363	371	0.055	27.7
Peptide_1	sp|P63128|POK9_HUMAN	100.000	9	0	0	1	9	363	371	0.12	26.6
Peptide_1	sp|Q9HDB9|GAK5_HUMAN	88.889	9	1	0	1	9	363	371	0.16	26.2
Peptide_2	sp|P87889|GAK10_HUMAN	100.000	9	0	0	1	9	344	352	0.34	25.4
Peptide_2	sp|P62684|GA113_HUMAN	100.000	9	0	0	1	9	344	352	0.36	25.4
Peptide_2	sp|P63130|GAK7_HUMAN	100.000	9	0	0	1	9	344	352	0.36	25.4
Peptide_2	sp|Q7LDI9|GAK6_HUMAN	100.000	9	0	0	1	9	344	352	0.38	25.0
Peptide_2	sp|P63145|GAK24_HUMAN	100.000	9	0	0	1	9	344	352	0.38	25.0
Peptide_2	sp|P62683|GAK21_HUMAN	100.000	9	0	0	1	9	344	352	0.38	25.0
Peptide_2	sp|Q9YNA8|GAK19_HUMAN	100.000	9	0	0	1	9	344	352	0.39	25.0
Peptide_2	sp|P63126|GAK9_HUMAN	100.000	9	0	0	1	9	344	352	0.39	25.0
Peptide_2	sp|P62685|GAK8_HUMAN	100.000	9	0	0	1	9	344	352	0.39	25.0
Peptide_2	sp|P63128|POK9_HUMAN	100.000	9	0	0	1	9	344	352	0.59	24.6
Peptide_2	sp|Q9HDB9|GAK5_HUMAN	87.500	8	1	0	1	8	344	351	4.0	22.3
Peptide_3	sp|P63135|POK7_HUMAN	100.000	9	0	0	1	9	208	216	0.57	24.6
Peptide_3	sp|P10266|POK10_HUMAN	100.000	9	0	0	1	9	208	216	0.58	24.6
Peptide_3	sp|Q9UQG0|POK11_HUMAN	100.000	9	0	0	1	9	208	216	0.58	24.6
Peptide_3	sp|Q9WJR5|POK19_HUMAN	100.000	9	0	0	1	9	211	219	0.58	24.6
Peptide_3	sp|P63133|POK8_HUMAN	100.000	9	0	0	1	9	208	216	0.58	24.6
Peptide_3	sp|P63132|PO113_HUMAN	100.000	9	0	0	1	9	208	216	0.58	24.6
Peptide_3	sp|Q9BXR3|POK6_HUMAN	100.000	9	0	0	1	9	208	216	0.58	24.6
Peptide_3	sp|P63136|POK25_HUMAN	100.000	9	0	0	1	9	208	216	0.58	24.6
Peptide_3	sp|Q9QC07|POK18_HUMAN	100.000	9	0	0	1	9	208	216	0.58	24.6
Peptide_4	sp|P63128|POK9_HUMAN	100.000	9	0	0	1	9	15	23	6.5	21.6
Peptide_4	sp|Q9HDB9|GAK5_HUMAN	100.000	9	0	0	1	9	15	23	6.6	21.6
Peptide_4	sp|P63145|GAK24_HUMAN	100.000	9	0	0	1	9	15	23	6.6	21.6
Peptide_4	sp|P63130|GAK7_HUMAN	100.000	9	0	0	1	9	15	23	6.6	21.6
Peptide_4	sp|P63126|GAK9_HUMAN	100.000	9	0	0	1	9	15	23	6.6	21.6
Peptide_4	sp|P62684|GA113_HUMAN	100.000	9	0	0	1	9	15	23	6.6	21.6
Peptide_4	sp|Q9YNA8|GAK19_HUMAN	100.000	9	0	0	1	9	15	23	6.6	21.6
Peptide_4	sp|P62683|GAK21_HUMAN	100.000	9	0	0	1	9	15	23	6.6	21.6
Peptide_4	sp|P87889|GAK10_HUMAN	100.000	9	0	0	1	9	15	23	6.6	21.6
Peptide_4	sp|Q7LDI9|GAK6_HUMAN	100.000	9	0	0	1	9	15	23	6.6	21.6
Peptide_4	sp|P62685|GAK8_HUMAN	100.000	9	0	0	1	9	15	23	6.6	21.6
Peptide_5	sp|Q9WJR5|POK19_HUMAN	100.000	9	0	0	1	9	113	121	0.76	24.3
Peptide_5	sp|Q9QC07|POK18_HUMAN	100.000	9	0	0	1	9	110	118	0.76	24.3
Peptide_5	sp|Q9BXR3|POK6_HUMAN	100.000	9	0	0	1	9	110	118	0.77	24.3
Peptide_5	sp|P63133|POK8_HUMAN	100.000	9	0	0	1	9	110	118	0.79	24.3
Peptide_5	sp|P63136|POK25_HUMAN	100.000	9	0	0	1	9	110	118	0.80	24.3
Peptide_5	sp|P63132|PO113_HUMAN	100.000	9	0	0	1	9	110	118	0.82	24.3
Peptide_5	sp|P63135|POK7_HUMAN	100.000	9	0	0	1	9	110	118	1.2	23.9
Peptide_5	sp|Q9UQG0|POK11_HUMAN	100.000	9	0	0	1	9	110	118	1.3	23.5
Peptide_5	sp|P10266|POK10_HUMAN	100.000	9	0	0	1	9	110	118	1.7	23.5
Peptide_5	sp|P63128|POK9_HUMAN	100.000	9	0	0	1	9	1033	1041	4.3	22.3
Peptide_6	sp|P63135|POK7_HUMAN	100.000	9	0	0	1	9	287	295	2.6	22.7
Peptide_6	sp|P10266|POK10_HUMAN	100.000	9	0	0	1	9	287	295	2.7	22.7
Peptide_6	sp|Q9UQG0|POK11_HUMAN	100.000	9	0	0	1	9	287	295	2.7	22.7
Peptide_6	sp|Q9WJR5|POK19_HUMAN	100.000	9	0	0	1	9	290	298	2.7	22.7
Peptide_6	sp|P63133|POK8_HUMAN	100.000	9	0	0	1	9	287	295	2.7	22.7
Peptide_6	sp|Q9BXR3|POK6_HUMAN	100.000	9	0	0	1	9	287	295	2.7	22.7
Peptide_6	sp|P63136|POK25_HUMAN	100.000	9	0	0	1	9	287	295	2.7	22.7
Peptide_6	sp|Q9QC07|POK18_HUMAN	100.000	9	0	0	1	9	287	295	2.7	22.7

Table S2: Blast results of the 6 HERV peptides aligned against the human proteome.

Peptide	HLA-A0101	HLA-A0201	HLA-A0301	HLA-A2402	HLA-B0702	HLA-B1501	HLA-B2705	HLA-B4401	HLA-B5801
FLQFKTWWI	5.7	0.04	8.61	2.51	8.76	9.82	23.36	22.26	7.4
RLIPYDWEI	11.76	0.03	4.13	0.86	9.14	2.29	7.51	8.98	1.22
KLIDCYTFL	5.22	0.02	1.91	0.61	4.66	1.31	7.57	12.88	1.34
YLSFIKILL	4.95	0.05	6.09	1.56	3.74	2.7	3	16.28	4.13
AMIPKDWPL	13.05	0.05	4.54	1.36	2.53	0.86	8.56	12.26	3.58
YAMSNLFSI	6.16	0.05	7.42	0.85	1.63	2.18	2.94	8.22	0.51

	Strong binder
	Weak Binder

Table S3: Prediction of binding for the most frequent HLA-A and B alleles. Strong binders (rank <= 0.5 percentile) and weak binders (rank between 0.5 and 2 percentiles) are highlighted in yellow and green, respectively.

Peptide	peptide sequence	Corresponding HERV sequences	HERV antigen	priming assay	SLP processing	Mass spectrometry detection	co-culture T2 (intracellular cytokine staining)	clones and TCR modeling	clone avidity	cytotoxicity clones vs. Cell lines	cytotoxicity clones vs. Organoids	Specific cells found among TILs
P1	FLQFKTWWI	*herv_483, herv_6069, herv_2025, herv_2704, herv_3192, herv_2582, herv_4695, herv_3652*	*Gag*	+	+	+	+	+	+	+	+	+
P2	RLIPYDWEI	*herv_4833, herv_6069, herv_2025, herv_6079, herv_2704, herv_3192, herv_2582, herv_4695herv_3652*	*Gag*	+	+		+	+	+	+		+
P3	KLIDCYTFL	*herv_4873, herv_6069, herv_2025, herv_1741, herv_2582, herv_4695, herv_3652*	*Pol*	+			+					
P4	YLSFIKILL	*herv_4873, herv_6069, herv_2025, herv_6079, herv_2704, herv_1741, herv_3192, herv_2582, herv_4695*	*Gag*	+			+	+				+
P5	AMIPKDWPL	*herv_4873, herv_6069, herv_2025, herv_6079, herv_2704, herv_2794, herv_2582, herv_4695, herv_3652*	*Pol*	+			+					+
P6	YAMSNLFSI	*herv_4873, herv_6069, herv_2025, herv_6079, herv_2704, herv_2794, herv_2582, herv_4695, herv_3652*	*Pol*	+	+	+	+	+	+	+	+	+

Table S4: Summary of the results obtained with each epitope. Validated steps are indicated with “+”. Empty boxes indicate non performed/non validated steps.

Movie S1.

3D microscopy imaging using Nanolive technology of P1- specific T cells co-cultured with MDA-MB-231 cells

Movie S2.

3D microscopy imaging using Nanolive technology of P1- specific T cells co-cultured with HMEC cells

Movie S3.

3D microscopy imaging using Nanolive technology of dextramer-negative non-specific T cells co-cultured with MDA-MB-231 cells

Movie S4.

3D microscopy imaging using Nanolive technology of P6-specific T cells co-cultured with HCC1599 cells

Movie S5.

3D microscopy imaging using Nanolive technology of dextramer-negative non-specific T cells co-cultured with HCC1599 cells
